# Establishment of the lymphoid ETS-code reveals deregulated ETS genes in Hodgkin lymphoma

**DOI:** 10.1371/journal.pone.0288031

**Published:** 2023-07-10

**Authors:** Stefan Nagel, Corinna Meyer, Claudia Pommerenke

**Affiliations:** Department of Human and Animal Cell Lines, Leibniz-Institute DSMZ, German Collection of Microorganisms and Cell Cultures, Braunschweig, Germany; Universitat des Saarlandes, GERMANY

## Abstract

The human family of ETS transcription factors numbers 28 genes which control multiple aspects of development, notably the differentiation of blood and immune cells. Otherwise, aberrant expression of ETS genes is reportedly involved in forming leukemia and lymphoma. Here, we comprehensively mapped ETS gene activities in early hematopoiesis, lymphopoiesis and all mature types of lymphocytes using public datasets. We have termed the generated gene expression pattern lymphoid ETS-code. This code enabled identification of deregulated ETS genes in patients with lymphoid malignancies, revealing 12 aberrantly expressed members in Hodgkin lymphoma (HL). For one of these, ETS gene ETV3, expression in stem and progenitor cells in addition to that in developing and mature T-cells was mapped together with downregulation in B-cell differentiation. In contrast, subsets of HL patients aberrantly overexpressed ETV3, indicating oncogenic activity in this B-cell malignancy. Analysis of ETV3-overexpressing HL cell line SUP-HD1 demonstrated genomic duplication of the ETV3 locus at 1q23, GATA3 as mutual activator, and suppressed BMP-signalling as mutual downstream effect. Additional examination of the neighboring ETS genes ETS1 and FLI1 revealed physiological activities in B-cell development and aberrant downregulation in HL patient subsets. SUP-HD1 showed genomic loss on chromosome 11, del(11)(q22q25), targeting both ETS1 and FLI1, underlying their downregulation. Furthermore, in the same cell line we identified PBX1-mediated overexpression of RIOK2 which inhibited ETS1 and activated JAK2 expression. Collectively, we codified normal ETS gene activities in lymphopoiesis and identified oncogenic ETS members in HL.

## Introduction

In the course of hematopoiesis, production of the different types of blood and im-mune cells follows a tree-like system. Hematopoietic stem cells (HSCs) residing in the bone marrow, generate common lymphoid and common myeloid progenitors (CLPs and CMPs), the starting points for lymphopoiesis and myelopoiesis, respectively. The lymphoid lineage produces B-cells, T-cells, NK-cells and innate lymphoid cells (ILCs). In contrast to NK-cells and ILCs, differentiation of B-cells and T-cells terminates outside the bone marrow in lymph nodes and the thymus, respectively. All these differentiation steps are controlled at the transcriptional level [1,2]. Therefore, understanding normal and abnormal regulation of hematopoiesis requires knowledge of transcription factors (TFs), representing the main players.

The human genome encodes about 1600 TFs [3]. These are classified according to similarities in sequence and structure of their DNA-binding domains. Important TF families controlling hematopoiesis include basic helix-loop-helix, ETS, homeodomain, T-box, and Zinc-finger factors [4]. For comprehensive descriptions of active TFs in the hematopoietic compartment, we have mapped selected TF families to generate so-called TF-codes. According to that approach, we have reported the NKL-code for NKL homeobox genes, the TALE-code for TALE-class homeobox genes, and the TBX-code for T-box genes [5–8]. By describing physiological conditions of TFs activated in all stages of hematopoiesis, these codes enable deregulated TF-encoding genes in lymphoid and myeloid malignancies to be identified and assessed.

In this study, we focused on the ETS family of TFs [9,10]. Their nomenclature derives from an avian erythroblastosis virus which carries the oncogene E-twenty-six [11]. The human genome encodes 28 ETS factors. These share the about 85 amino acids long ETS domain which consists of three alpha-helices and a four-stranded antiparallel beta-sheet scaffold, and thus belongs to the winged helix-turn-helix group of TFs [12,13]. Differences in ETS domain sequences allow classification of these factors into nine subfamilies [10]. Functionally, this domain performs both DNA-binding and protein-interaction [14]. DNA-sequences recognized by particular ETS factors show only limited differences, indicating that these TFs control specific gene activities in concert with cofactors [15,16].

Several ETS factors are reportedly involved in regulation of hematopoietic devel-opment, including ETS1, FLI1, ETV6/TEL, SPI1/PU.1, and SPIB [17]. ETS1, for example, interacts with master factor PAX5 to regulate B-cell specific target genes [18,19]. On the other hand, mutated or deregulated ETS factors have been described in cancer, notably leukemia and lymphoma [20]. ETV6, for example, is part of diverse oncogenic fusion genes in lymphoid and myeloid malignancies while FLI1 operates context-dependently either as oncogene or tumor suppressor gene [20,21].

Over the years, classification of lymphoid neoplasms has been steadily modified and recently revised [22]. According to this system, Hodgkin lymphoma (HL) is a B-cell malignancy separated into classical HL and nodular lymphocyte predominant HL (NLPHL). Pathologically, HL tumor cells share aberrant survival by activation of NFkB-signalling while downregulation of several master factors controlling B-cell differentiation, including PAX5 and EBF1 is restricted to classical HL [23–25].

Here, we have generated the lymphoid ETS-code, delineating physiological activities of ETS genes in lymphopoiesis. Subsequent evaluation of ETS genes using public expression data from HL patients revealed several deregulated members including ETV3, ETS1 and FLI1 analyzed in more detail using HL cell lines as models.

## Results

### Establishment of the lymphoid ETS-code

**Fig 1:**
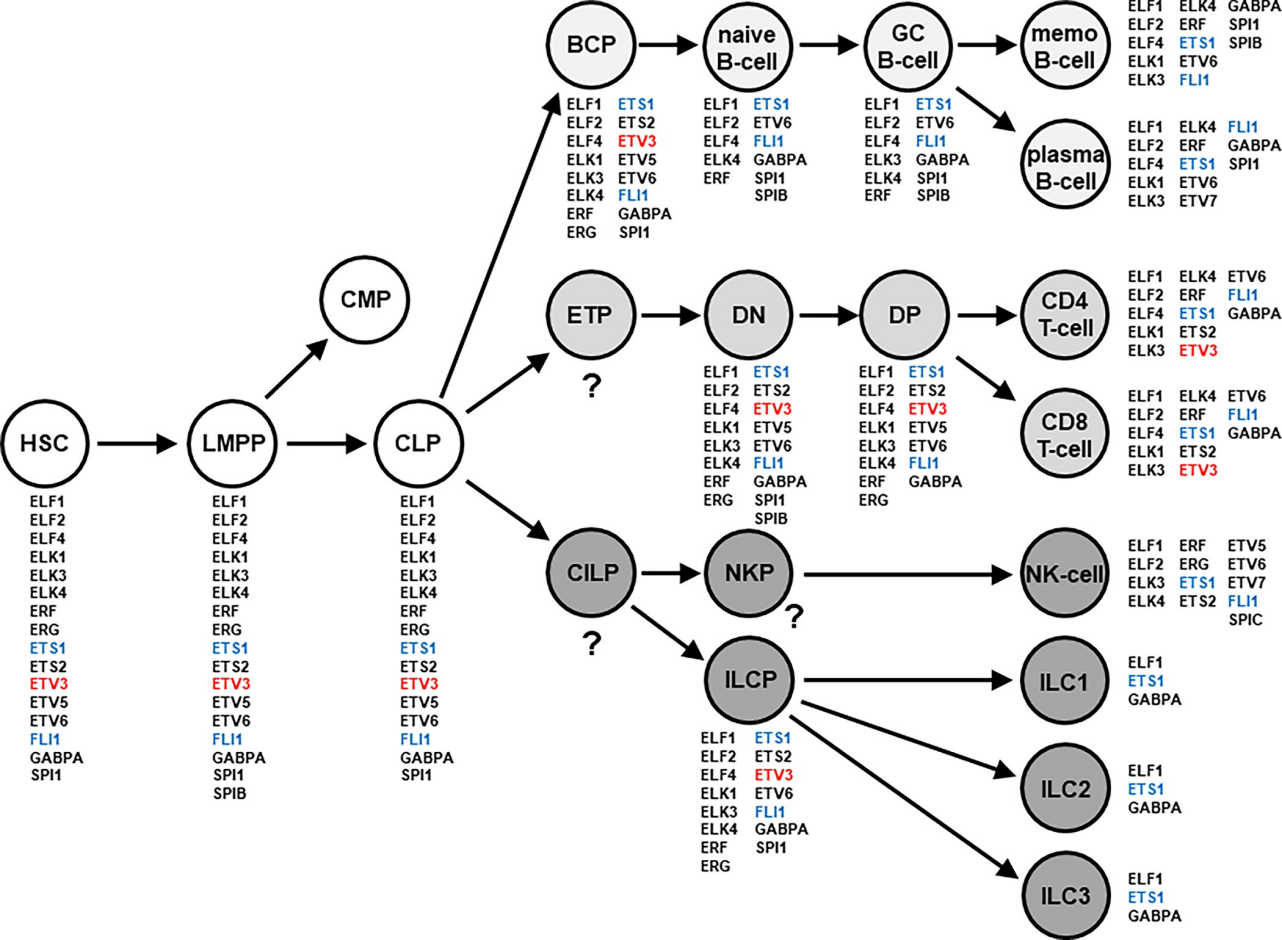
Graphical representation of the lymphoid ETS-code, showing ETS gene actvities in entities from early hematopoiesis and lymphopoiesis. This code was generated using public datasets. Three ETS genes are highlighted: ETV3 in red, and ETS1 and FLI1 in blue. Abbreviations: BCP: B-cell progenitor, CILP: Common innate lymphoid progenitor, CLP: Common lymphoid progenitor, CMP: Common myeloid progenitor, DN: Double negative T-cell, DP: Double positive T-cell, ETP: Early T-cell progenitor, GC: Germinal center, HSC: Hematopoietic stem cell, ILC: Innate lymphoid cell, ILCP: Innate lymphoid cell progenitor, LMPP: Lymphoid and myeloid primed progenitor, memo: Memory, NKP: NK-cell progenitor.

By exploiting several public datasets containing gene expression profiling or RNA-sequencing data, we have mapped the activities of all 28 ETS genes in normal hu-man hematopoietic entities. This approach corresponded with previously reported screenings [5–8]. We analyzed datasets for early hematopoiesis including stem and progenitor cells, for developing and mature T-cells, B-cells, and ILCs, in addition to comprehensive RNA-seq data covering a variety of cells from several normal hematopoietic lineages (**S1–S4 Figs**). The combined results are given in **Fig 1**, representing the so-called lymphoid ETS-code. According to this code, we detected 19 ETS genes expressed in early hematopoiesis and lymphopoiesis, including ELF1, ELF2, ELF4, ELK1, ELK3, ELK4, ERF, ERG, ETS1, ETS2, ETV3, ETV5, ETV6, ETV7, FLI1, GABPA, SPI1, SPIB and SPIC, ranging from 3 to 17 genes per entity.

Inspection of their activities revealed interesting assignments: ELF1 and ETS1 were active in all entities, GABPA remained silent in NK-cells only, and ERF was expressed ubiquitously except in ILCs. ETV5 was active in progenitors and silent in mature cells, while SPIC expression was restricted to NK-cells. ERG was expressed in progenitor cells and mature NK-cells but downregulated in B-cells, T-cells, and ILCs. Finally, ELK1 showed activity in all entities except NK-cells and ILCs. Looking at developing B-cells, the data indicated that ETV7 was active in plasma cells but silent in memory B-cells while SPIB was expressed in memory B-cells but repressed in plasma cells. ETV3 was found to be downregulated in the development of B-cell while still active in that of T-cells. Alltogether, this code showed several conspicuous gene expression patterns, probably underlying specific differentiation processes or the maintenance of immune cell identities.

### Deregulated ETS genes in Hodgkin lymphoma

The established lymphoid ETS-code assists identification of deregulated ETS genes in lymphoid malignancies. Here, we analyzed HL using the public gene expression profiling dataset GSE12453 which contains 17 HL patients in addition to other types of lymphoma, and normal developing B-cells, serving as controls (**S5 Fig**). According to this approach, we compended deregulated ETS genes in HL patients as shown in **Table 1**, detecting seven aberrantly upregulated (EHF, ELK1, ETS2, ETV3, ETV6, ETV7, SPIC) and five downregulated ETS genes (ELF1, ELF2, ELK3, ETS1, FLI1). Normally, ETV3 remained active in T-cell development while downregulated in the development of B-cells and ILCs, and the activity of SPIC was restricted to NK-cells. Thus, aberrant expression of these genes may disturb processes of normal B-cell differentiation by reactivating programs appropriated from other lineages, or by stopping the normal B-cell program. ETS1 and FLI1 were widely expressed in lymphopoiesis. Their aberrant downregulation may, therefore, affect rather basic aspects of lymphoid differentiation.

**Table 1:** Aberrant ETS gene activities in HL patients and cell lines.

gene	ID	HL patients (GSE12453)	HL cell lines (LL-100: E-MTAB-7721)
**EHF**	225645_at	up	L-428
**ELF1**	212418_at	down	L-428, L-1236, SUP-HD1
**ELF2**	203822_s_at	down	SUP-HD1
**ELF3**	210827_s_at		
**ELF4**	31845_at		
**ELF5**	220625_s_at		
**ELK1**	203617_x_at	up	---
**ELK3**	221773_at	down	L-428, L-1236, SUP-HD1
**ELK4**	205994_at		
**ERF**	203643_at		
**ERG**	213541_s_at		
**ETS1**	224833_at	down	SUP-HD1
**ETS2**	201328_at	up	SUP-HD1
**ETV1**	206501_x_at		
**ETV2**	215510_at		
**ETV3**	1552423_at	up	HDLM-2, KM-H2, L-1236, SUP-HD1
**ETV3L**	-	?	
**ETV4**	1554576_a_at		
**ETV5**	203349_s_at		
**ETV6**	235056_at	up	HDLM-2
**ETV7**	221680_s_at	up	HDLM-2
**FEV**	207260_at		
**FLI1**	204236_at	down	SUP-HD1
**GABPA**	210188_at		
**SPDEF**	213441_x_at		
**SPI1**	205312_at		
**SPIB**	205861_at		
**SPIC**	1553851_at	up	DEV

Cell lines are suitable models to analyze regulation and function of genes in the context of their derived malignancy [26]. Therefore, we screened the LL-100 RNA-seq dataset (E-MTAB-7721) containing 100 hematopoietic cell lines derived from myeloid and lymphoid types of leukemia and lymphoma, including HL [27]. HL cell lines showing overexpression or downregulation of those ETS genes identified in HL patients were included in **Table 1** (**S6 Fig**). Thus, we found for each ETS gene deregulated in HL patients one or more corresponding HL cell lines to serve as models for detailed analyses.

### ETV3 in Hodgkin lymphoma

**Fig 2:**
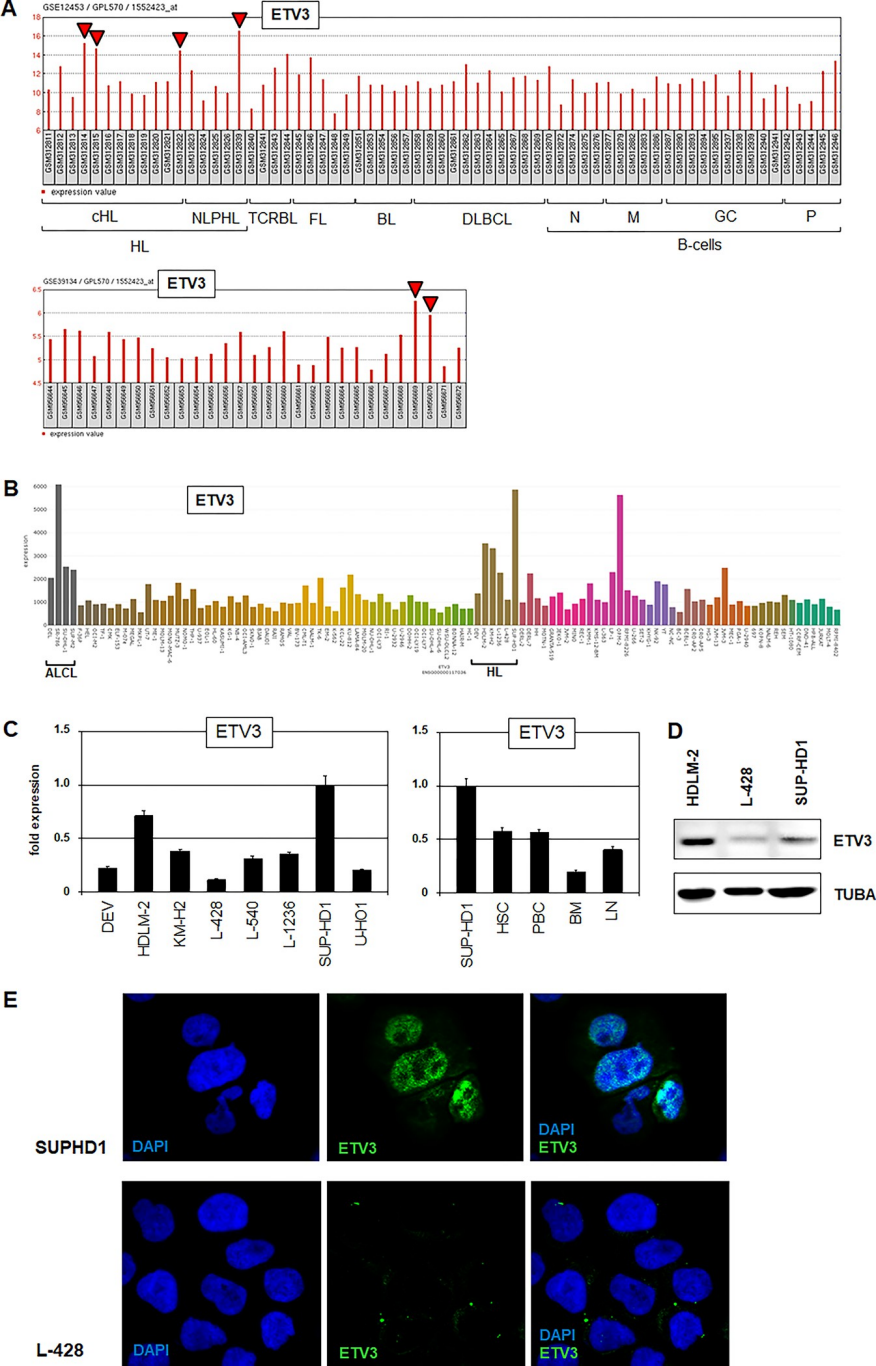
ETV3 expression in HL patients and cell lines. (A) Expression profiling data (dataset GSE12543, above) for ETV3 in patients with HL, T-cell rich B-cell lymphoma (TCRBL), follicular lym-phoma (FL), Burkitt lymphoma (BL), diffuse large B-cell lymphoma (DLBCL), and normal B-cells: Naïve B-cells (N), memory B-cells (M), germinal center B-cells (GC), plasma cells (P), and dataset GSE39134 with classical HL patients (below). HL patients showing ETV3 overexpression are indicated by arrow heads. (B) ETV3 expression in leukemia/lymphoma cell lines using RNA-seq dataset LL-100. ALCL and HL cell lines are indicated. (C) RQ-PCR expression analysis of ETV3 in HL cell lines (left) and primary cells (right): Hematopoietic stem cell (HSC), peripheral blood cells (PBC), bone marrow (BM), lymph node (LN). (D) Western blot analysis of ETV3 and TUBA in HL cell lines. (E) Immuno-staining of ETV3 (green) in HL cell lines SUP-HD1 (above) and L-428 (below). DAPI (blue) served as nuclear counterstain.

In the following, we focused on ETS gene ETV3, downregulated in normal B-cell development (**Fig 1**) and aberrantly activated in 4/17 (23%) HL patients of dataset GSE12453 including classical HL and NLPHL, and in 2/29 (7%) classical HL patients of dataset GSE39134 (**Fig 2A**). The LL-100 RNA-seq dataset showed elevated ETV3 expression in 4/6 HL cell lines and additionally in 4/4 cell lines derived from anaplastic large cell lymphoma (ALCL) (**Fig 2B**). This observation may be of interest because HL and ALCL share some pathological features [28]. RQ-PCR and Western blot analysis confirmed peak ETV3 expression levels in HL cell lines HDLM-2 and SUP-HD1 (**Fig 2C and 2D**). Of note, elevated protein expression levels in HDLM-2 may indicate additional post-transcriptional regulation. Furthermore, ETV3 expression in SUP-HD1 was higher when compared to primary samples from selected normal hematopoietic cells and tissues (**Fig 2C**). Finally, immunostaining of ETV3 showed nuclear localization in SUP-HD1 and absent protein expression in L-428 (**Fig 2E**), supporting its reported function as TF, and attesting SUP-HD1 as suitable model to study the role of ETV3 in HL.

**Fig 3:**
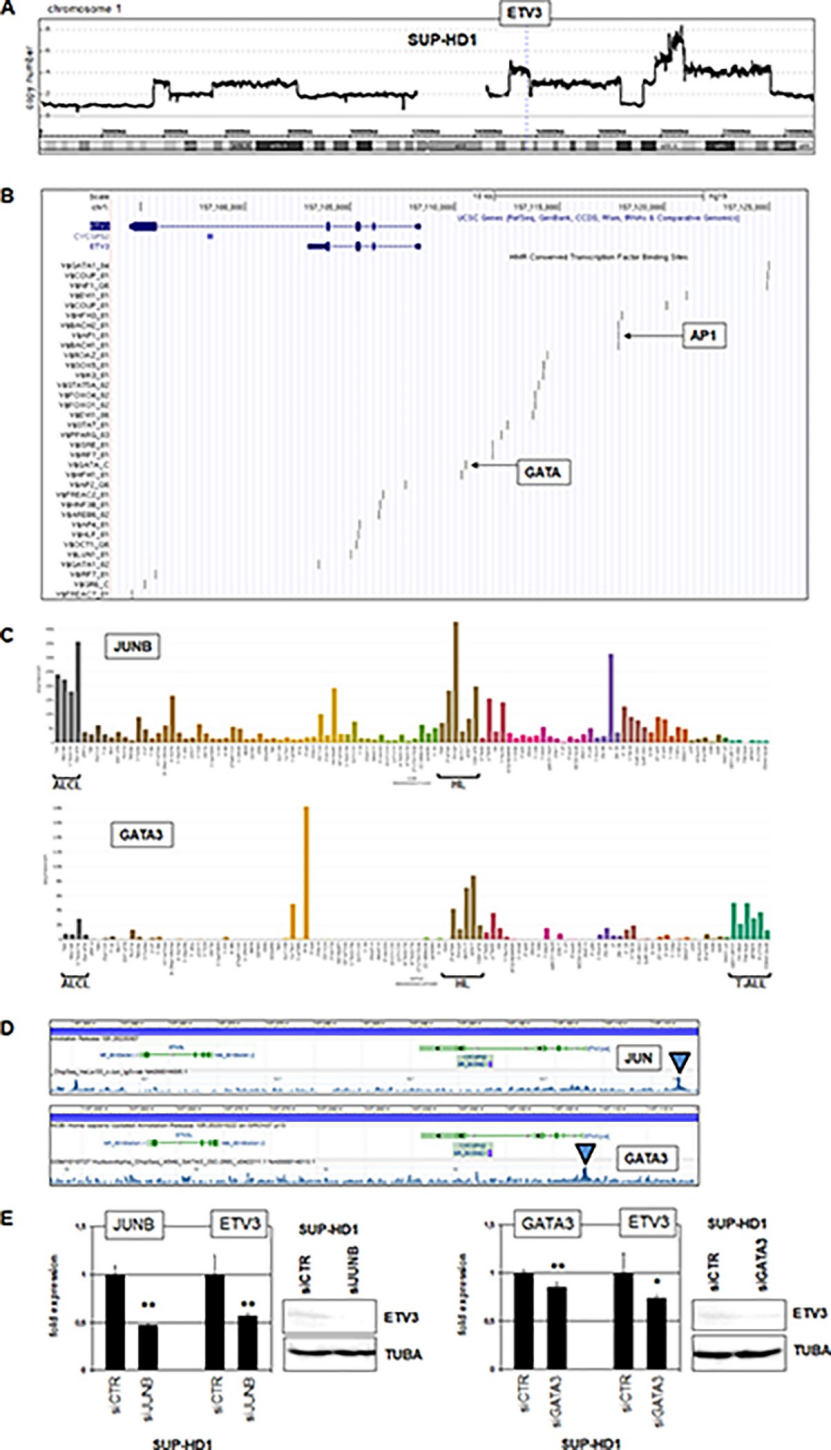
Mechanisms of aberrant ETV3 activation in HL. (A) Copy number data for chromosome 1 from HL cell line SUP-HD1. The ETV3 locus at 1q23 is indicated and targeted by a genomic duplication. (B) Potential TF-binding sites at ETV3 obtained from the UCSC genome browser. Binding sites for GATA and AP1 are indicated. (C) RNA-seq expression data for JUNB (above) and GATA3 (below) obtained from the LL-100 dataset. Cell lines derived from ALCL, HL, and T-ALL are indicated. (D) ChIP-seq data obtained from the ENCODE project, show binding of JUN (above) and GATA3 (below) in the promoter region of ETV3, corresponding to their indicated binding sites. (E) RQ-PCR and Western blot analyses of SUP-HD1 after treatment for siRNA-mediated knockdown of JUNB (left) and GATA3 (right), demonstrating their activating input in ETV3 expression.

Chromosomal rearrangements and copy number alterations may underlie gene de-regulation in tumor cells and frequently occur in HL [29–31]. The ETV3 locus is located on chromosome 1, band q23. However, inspection of reported karyotypes discounted rearrangements at 1q23 in HDLM-2 and SUP-HD1 [30,31]. In contrast, SUP-HD1 contained several copy number alterations at chromosome 1, including dup(1)(q23) which targeted ETV3 (**Fig 3A**).

Analysis of TF binding sites at ETV3 indicated potential regulatory impacts of AP1 and GATA (**Fig 3B**). AP1 factors JUN and JUNB, and zinc-finger factor GATA3 are reported oncogenes in HL (and additionally in ALCL), highlighting their potential role in ETV3 deregulation [32–35]. RNA-seq data demonstrated elevated expression of JUNB and GATA3 in HL and ALCL cell lines (**Fig 3C**). Moreover, public chromatin immuno-precipitation (ChIP)-seq data for JUN and GATA3 showed interaction at the promoter region of ETV3, corresponding to their indicated binding sites (**Fig 3D**). To test the impact of JUNB and GATA3 on ETV3 regulation, we performed siRNA-mediated knockdown experiments in SUP-HD1. The results demonstrated that both, JUNB and GATA3 activated ETV3 expression in HL cells (**Fig 3E**). Taken together, genomic duplication of the ETV3 locus and aberrant activities of JUNB and GATA3 contributed to deregulated expression of ETV3 in HL.

**Fig 4:**
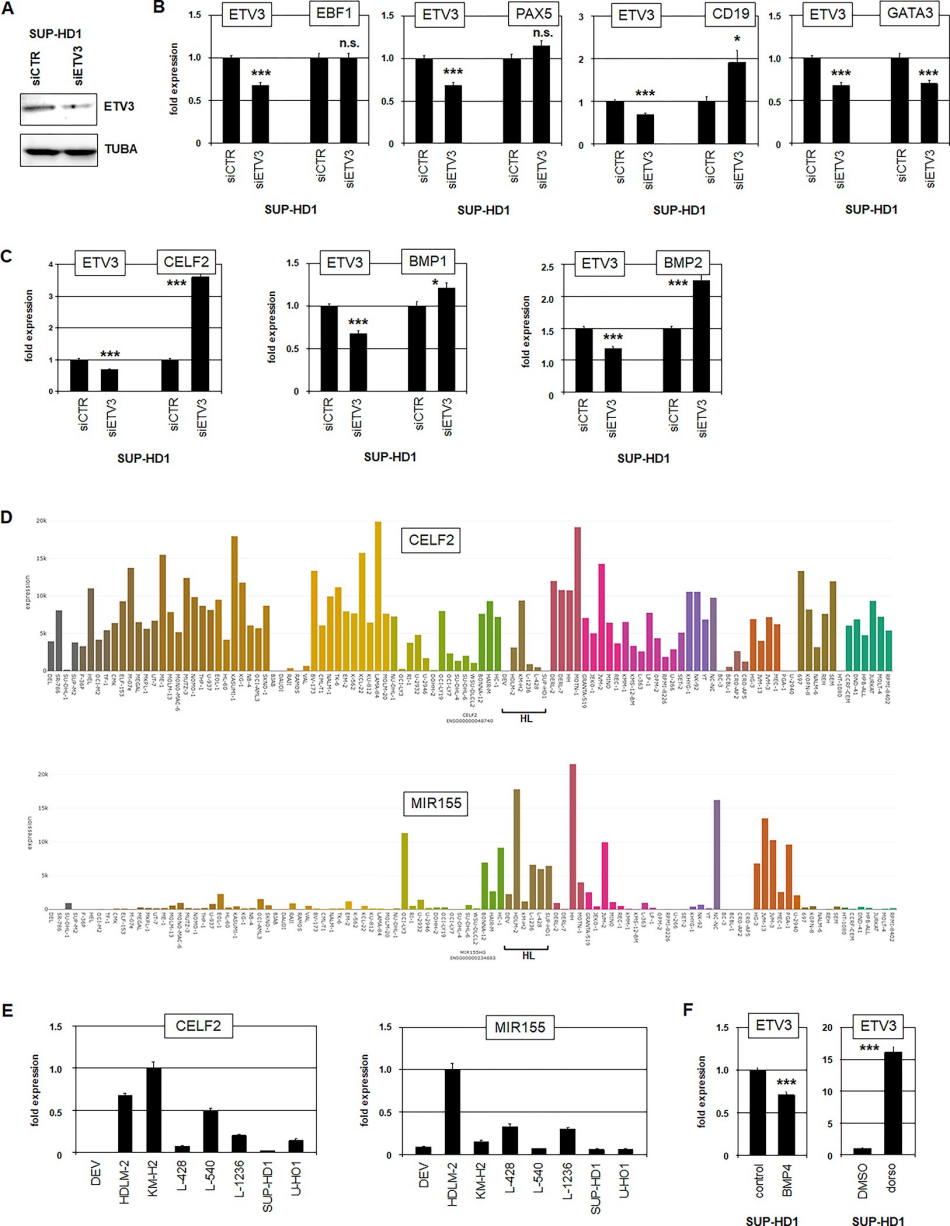
Target gene analyses of ETV3. (A) Western blot analysis of ETV3 and TUBA in SUP-HD1 after siRNA-mediated knockdown of ETV3. RQ-PCR analysis of EBF1, PAX5, CD19, and GATA3 (B), and of CELF2, BMP1, and BMP2 (C) in SUP-HD1 after siRNA-mediated knockdown of ETV3. (D) RNA-seq expression data for CELF2 (above) and MIR155 (below) obtained from the LL-100 dataset. Cell lines derived from HL are indicated. (E) RQ-PCR analysis of CELF2 (left) and MIR155 (right) in HL cell lines. (F) RQ-PCR analysis of ETV3 in SUP-HD1 after treatment with BMP4 (left) and BMP-inhibitor dorsomorphin (right).

Next, we examined the functional impact of ETV3 on gene regulation in HL cells. We used SUP-HD1 as model and performed siRNA-mediated knockdown of ETV3 which was confirmed by Western blot (**Fig 4A**) and RQ-PCR (**Fig 4B**). Given the reported role of ETS factors in cell and tissue differentiation, we analyzed regulation of genes encoding B-cell master factors EBF1 and PAX5, B-cell differentiation marker CD19, and T-cell master factor GATA3. Our data indicated absent impacts of ETV3 on EBF1 and PAX5 expression while CD19 was suppressed and GATA3 activated (**Fig 4B**), demonstrating ETV3 and GATA3 as mutual activators. Consistent with these findings, we detected an ETS-binding site in the upstreamregion of CD19 (**S7 Fig**), indicating a direct regulatory impact by ETV3.

Gene expression profiling analysis of SUP-HD1 cells treated for ETV3-knockdown revealed additional target gene candidates including suppression of miR155-repressor CELF2 (**S1 Table**). MIR155 is reportedly an oncogene overexpressed in HL [36]. Furthermore, we detected an ETS-binding site within the intronic region of CELF2 (**S7 Fig**), supporting the relevance of this potential regulation. Gene set annotation analysis of the top-1000 differentially expressed genes using the online tool DAVID indicated that ETV3 activated developmental processes (chondrocytes, kidney and bone), and suppressed BMP-signalling via BMP1 and BMP2 (**S8 Fig, S1 Table**).

In addition, RQ-PCR analysis of CELF2, BMP1 and BMP2 after treatment for siRNA-mediated knockdown in SUP-HD1 confirmed that ETV3 suppressed these genes (**Fig 4C**). In accordance with our results, gene expression data from the LL-100 dataset showed that HL cell lines expressed reduced levels of CELF2 along with elevated MIR155 (**Fig 4D**) which was confirmed by RQ-PCR analysis (**Fig 4E**). Moreover, HDLM-2 contained a genomic deletion nearby the locus of MIR155 at 21q21 which may contribute to its activation in this cell line (**S9 Fig**). Finally, treatment of SUP-HD1 with BMP4 and BMP-pathway inhibitor dorsomorphin demonstrated that BMP-signalling inhibited ETV3 expression (**Fig 4F**), indicating feedback regulation. Thus, our target gene analysis identified roles for ETV3 in (deregulated) differentiation processes, activation of miR155, and suppression of BMP-signalling.

**Fig 5:**
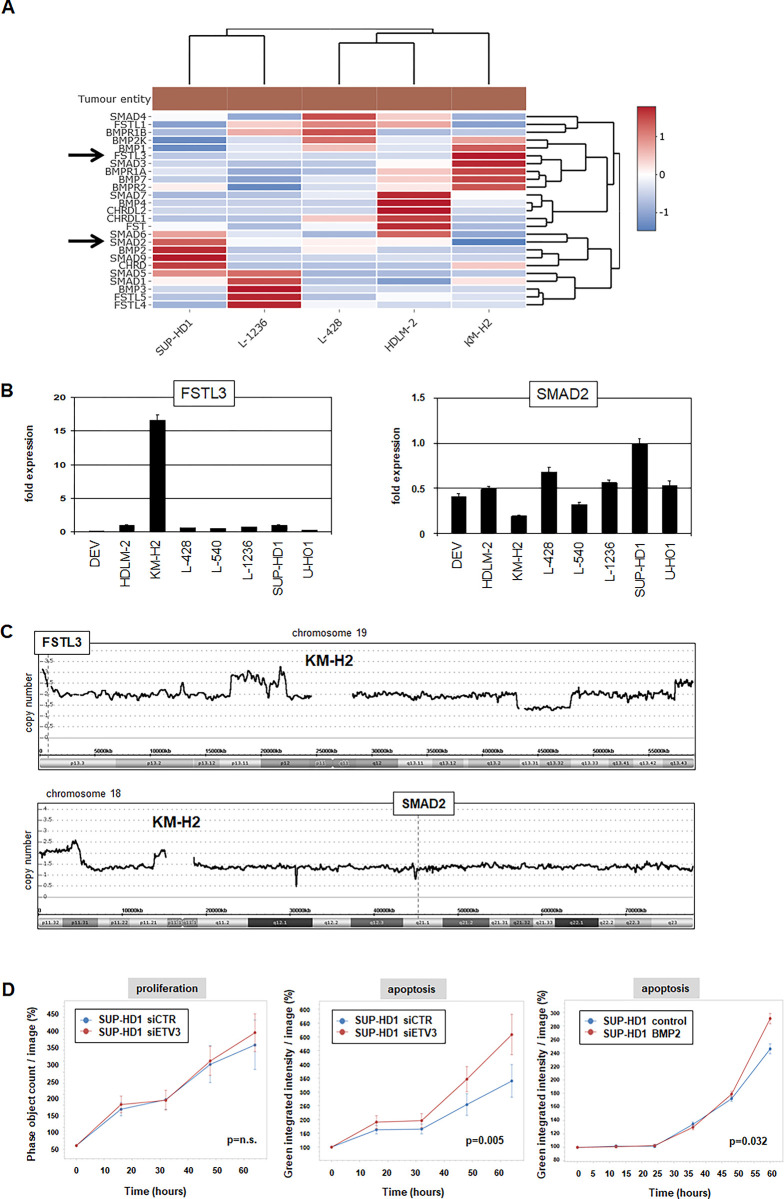
BMP-pathway analysis in HL. (A) Heat map showing gene expression data for five HL cell lines and selected components of the BMP-pathway obtained from the LL-100 RNA-seq dataset. Arrows indicate genes FSTL3 and SMAD2. (B) RQ-PCR expression analysis of FSTL3 and SMAD2 in HL cell lines. (C) Copy number data for chromosome 19 (above) and chromosome 18 (below) from cell line SUP-HD1. Gene locis FSTL3 at 19p13 and SMAD2 at 18q21 are indicated. (D) Live-cell imaging results for cell line SUP-HD1 following siRNA-mediated knockdown of ETV3 (left and middle) and with BMP2 (right). The indicated p-values refer to the last time point of treatment and control.

Our results showing that ETV3 inhibits BMP-signalling prompted us to address in more detail the role of this pathway in HL. An expression heatmap covering selected genes encoding components of the BMP-pathway in HL cell lines is shown in **Fig 5A**. The observed heterogeneity in expression levels indicated that in each cell line a different strategy to inhibit this pathway had been adopted. For example, KM-H2 expressed high levels of BMP-repressor FSTL3 and low levels of downstream effector SMAD2. These gene activities were confirmed by RQ-PCR analysis (**Fig 5B**), and correlated with copy number alterations at their loci in this cell line (**Fig 5C).**

Functional analyses of SUP-HD1 cells treated for ETV3-knockdown by live-cell-imaging indicated that ETV3 supports cell survival but without impacting cell proliferation (**Fig 5D**). Treatment of SUP-HD1 with BMP2 induced apoptosis (**Fig 5D**), demonstrating that inhibition of this pathway mediates cell survival. Collectively, our data revealed several modes of BMP-pathway inhibition in HL, including overexpression of FSTL3 and downregulation of SMAD2 via genomic alterations, and inhibition of BMPs via overexpressing ETV3. Suppression of BMP-signalling may, thus, represent an additional oncogenic cell survival mechanism in HL.

### ETS1 and FLI1 in Hodgkin lymphoma

**Fig 6:**
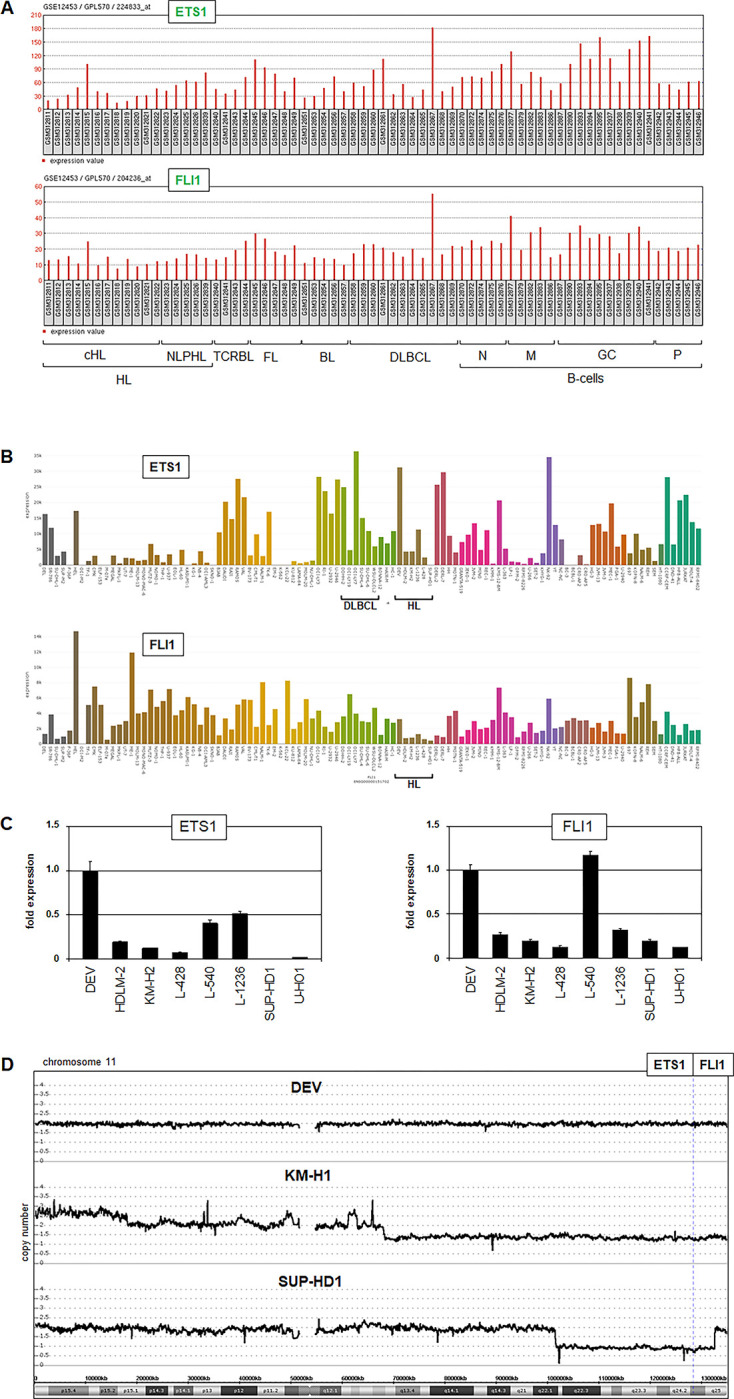
Reduced expression of ETS1 and FLI1 in HL. (A) Expression profiling data (dataset GSE12543) for ETS1 and FLI1 in patients with HL, T-cell rich B-cell lymphoma (TCRBL), follicular lymphoma (FL), Burkitt lymphoma (BL), diffuse large B-cell lymphoma (DLBCL), normal B-cells: Naïve B-cells (N), memory B-cells (M), germinal center B-cells (GC), plasma cells (P). (B) Expression of ETS1 and FLI1 in leukemia/lymphoma cell lines using RNA-seq dataset LL-100. Cell lines derived from HL and DLBCL are indicated. (C) RQ-PCR analysis of ETS1 (left) and FLI1 (right) in HL cell lines. (D). Copy number data for chromosome 11 from cell lines DEV, KM-H2, and SUP-HD1. The neighboring gene locis ETS1 and FLI1 at 11q24 are indicated.

Screening of ETS gene activities in HL patients and cell lines revealed several de-regulated members including aberrant downregulation of ETS1 and FLI1 (**Fig 6A and 6B**), though NLPHL cell line DEV rather showed overexpression of ETS1. RQ-PCR analysis of these genes in HL cell lines demonstrated lowest expression levels in SUP-HD1 while DEV and L-540 expressed elevated ETS1 and FLI1 transcripts (**Fig 6C**). ETS1 and FLI1 are genomic neighbors located at chromosome band 11q24. Our copy number data showed a genomic loss in SUP-HD1, del(11)(q22.1q25), covering both ETS genes. Furthermore, KM-H2 contained a more extended deletion at that region, del(11)(:q13.2→qter), while DEV showed normal copy numbers for the entire chromosome 11 (**Fig 6D**). Thus, in classical HL cell lines SUP-HD1 and KM-H2 copy number alterations correspond to suppression of ETS genes ETS1 and FLI1.

**Fig 7:**
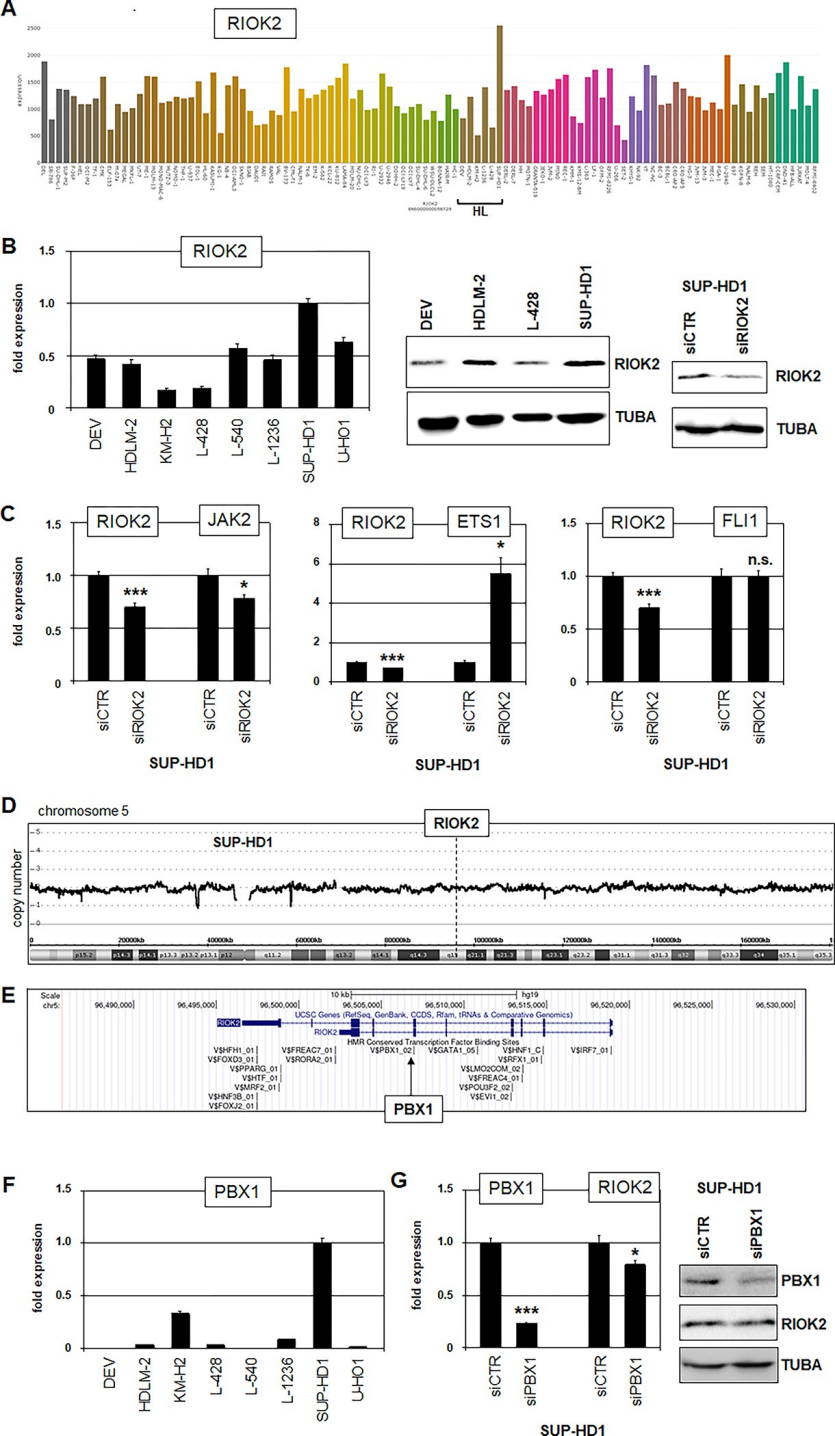
RIOK2 inhibits ETS1 in HL. (A) Expression of RIOK2 in leukemia/lymphoma cell lines using RNA-seq dataset LL-100. Cell lines derived from HL are indicated. (B) RQ-PCR analysis of RIOK2 in HL cell lines (left). Western blot analysis of RIOK2 and TUBA in HL cell lines and SUP-HD1 treated for RIOK2-knockdown (right). (C) RQ-PCR analysis of SUP-HD1 after treatment for siRNA-mediated knockdown of RIOK2, demonstrating an activating input in JAK2 and an inhibitory input in ETS1 expression while sparing FLI1. (D) Copy number data for chromosome 5 from cell line SUP-HD1. The RIOK2 locus at 5q15 is indicated. (E) Potential TF-binding sites at RIOK2, obtained from the UCSC genome browser. An intronic binding site for PBX1 is indicated. (F) RQ-PCR analysis of PBX1 expression in HL cell lines. (G) RQ-PCR analysis (left) and Western blot analysis (right) of RIOK2 in SUP-HD1 treated for siRNA-mediated knockdown of PBX1.

Recently, Ghosh and colleagues identified RIOK2 as master regulator of hemato-poiesis and showed that RIOK2 activates JAK2 and inhibits ETS1 and FLI1 [37]. Therefore, we addressed the role of RIOK2 in HL. RNA-seq data showed the highest RIOK2 expression in SUP-HD1 (**Fig 7A**), which was confirmed by RQ-PCR and Western blot analysis (**Fig 7B**). Of note, HDLM-2 showed elevated RIOK2 protein levels possibly indicating post-transcriptional regulation as mentioned before for ETV3. SiRNA-mediated knockdown experiments in SUP-HD1 demonstrated that RIOK2 activated JAK2 and inhibited ETS1 while sparing FLI1 (**Fig 7C**). Our copy number data gave no explanation for elevated RIOK2 expression in SUP-HD1 (**Fig 7D**), while JAK2 was amplified in both, HDLM-2 and SUP-HD1 (**S9 Fig**). However, TF binding-site analysis revealed a PBX1-site at RIOK2 (**Fig 7E**). PBX1 is reportedly aberrantly overexpressed in HL patients and cell line SUP-HD1 as shown in **Fig 7F** [6]. Knockdown of PBX1 resulted in reduced expression of RIOK2 (**Fig 7G**), demonstrating that PBX1 activated this gene in SUP-HD1. Thus, aberrantly elevated expression of RIOK2 is driven by overexpressed PBX1, mediating suppression of ETS1 in HL. Taken together, our data showed aberrant downregulation of ETS1 (and FLI1) in HL implemented by genomic deletion and RIOK2 overexpression.

## Discussion

**Fig 8:**
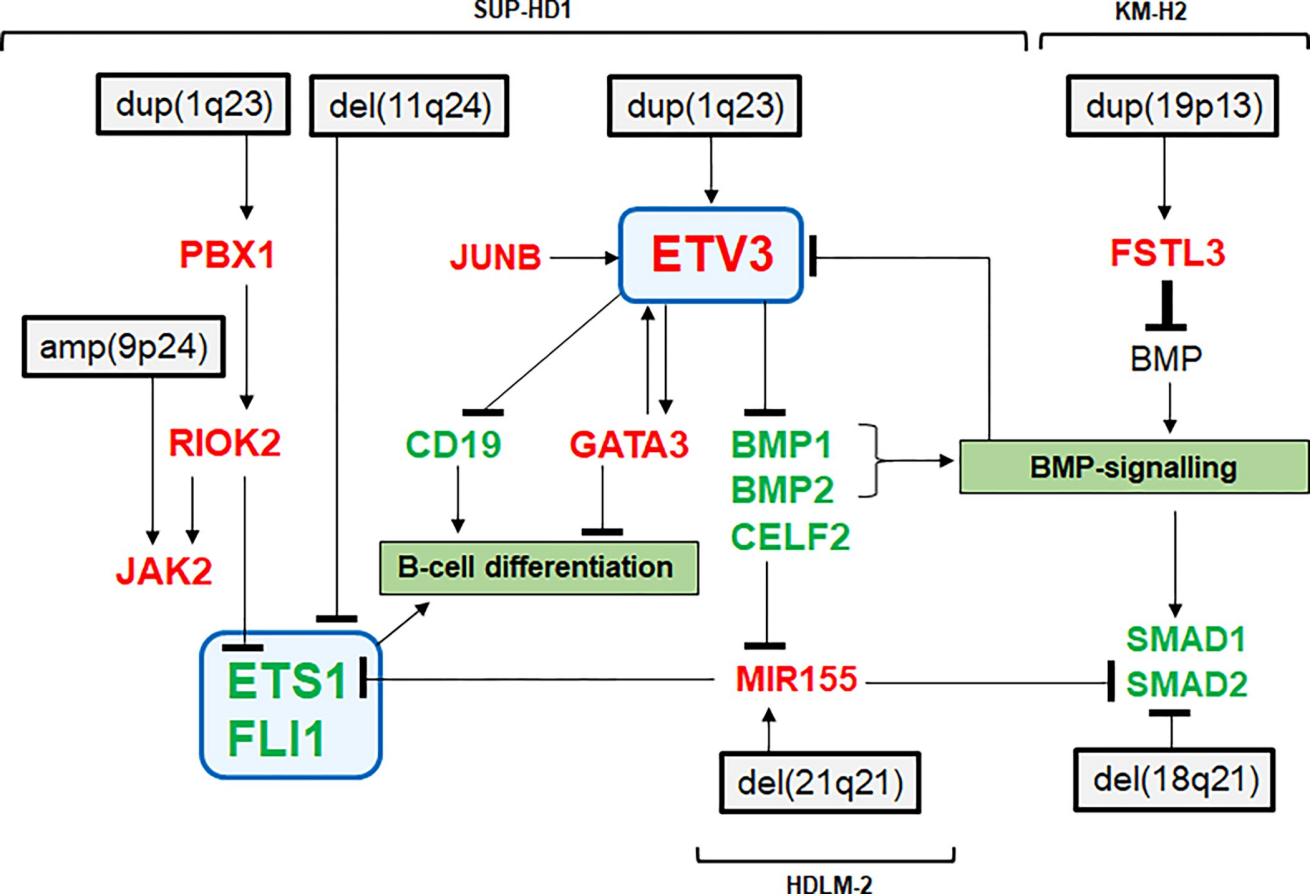
Summary of the results generated in this study combined with data from the literature. Deregulation of ETS factors ETV3, ETS1 and FLI1 are connected with inhibition of BMP-signalling and B-cell differentiation in HL and highlighted by a blue box. Overexpressed genes are indicated in red, downregulated in green.

We have previously established the concept of TF-codes which detail normal activi-ties of selected groups of TFs in the hematopoietic compartment and enable identification of deregulated TF-encoding genes in lymphoid and myeloid malignancies [5–8]. In this study, we have established the lymphoid ETS-code which describes physiological expression of 19 ETS genes in early hematopoiesis and lymphopoiesis. ETS genes encode developmental TFs showing specific expression patterns and may, thus, control differentiation processes in particular hematopoietic entities. Our data confirmed previously reported investigations of ETS factors, concerning for example ELF1, ETS1, FLI1, SPI1, and SPIB [17,38]. Of note, the ETS-code covers qualitative data and did not consider quantitative differences between genes and the entities characterized. Here, we analyzed regulation and function of the deregulated ETS genes ETV3, ETS1 and FLI1 in HL as summarized in **Fig 8**.

We showed that ETV3 represents a novel aberrantly expressed oncogene in HL. Normally, ETV3 was downregulated during B-cell development while genomic duplication and oncogenic activities of the TFs JUNB and GATA3 mediated aberrant ETV3 activation in HL cell line SUP-HD1. These factors may also underlie ETV3 activation in ALCL. Recently, amplification of ETV3 has been reported in breast cancer, supporting copy number gain as potent activating mechanism of this oncogene [39]. Detection of fusion gene ETV3::NCOA2 in independent cell histiocytosis (ICH) underlines a general oncogenic role for ETV3 in hematopoietic malignancies [40,41]. In the myeloid compartment, physiologically expressed ETV3 regulates macrophage differentiation [42,43], highlighting its functional role in developmental processes of myelopoiesis which may be aberrantly reactivated in HL subsets. Consistent with this picture, disturbed B-cell development via deregulated TFs is a recurrent oncogenic mechanism in the pathology of HL [23].

The BMP-pathway plays fundamental roles in controlling development, including normal hematopoiesis and B-cell differentiation, and in leukemia/lymphoma if deregulated [44–47]. Recently, we have shown that aberrantly expressed CHRDL1 promoted inhibition of BMP-signalling resulting in activation of NKL homeobox oncogene MSX1 in T-ALL [48]. Here, we demonstrated that downregulated BMP-signalling activated ETS gene ETV3 in HL. Thus, suppressed BMP-signalling frequently activates developmental TF-encoding genes in lymphoid malignancies. Our data indicated inhibition of BMP activity by ETV3 at the transcriptional level and by FSTL3 at the protein level. Furthermore, the FSTL3 locus was amplified in KM-H2 while that of SMAD2 showed a small genomic deletion nearby, targeting its regulatory region. Downregulation of the BMP-effector SMAD2 in HL also occurs via Epstein-Barr-virus (EBV)-infection [49]. Moreover, MIR155 is overexpressed in HL and targets SMAD2 as well [36,50], supporting the role of SMAD2 as a tumor suppressor in this malignancy. We have shown that CELF2 was downregulated by ETV3. CELF2 is a reported inhibitor of miR155 [51]. Thus, ETV3 promoted inhibition of BMP-signalling via suppression of both, BMPs and SMAD2 (**Fig 8**).

In addition to upregulation of ETS gene ETV3, we detected and analyzed downreg-ulation of ETS1 (and FLI1) in HL via genomic deletion which confirmed and detailed data from a recent study [52]. Furthermore, ETS1 was downregulated in SUP-HD1 by overexpressed RIOK2. RIOK2 encodes a kinase which represents a novel player in hematopoiesis and regulates several differentiation-related genes, including ETS1, FLI1 and JAK2 [37]. We showed, that the transcription of RIOK2 was activated by PBX1 which, in turn, is aberrantly overexpressed in HL patients and in cell line model SUP-HD1 [6]. Furthermore, ETS1 is a target of miR155 [53], thus representing an additional mechanism of its downregulation. In contrast to HL, ETS1 and FLI1 are aberrantly upregulated in subsets of DLBCL [54], pointing to differences in the pathogenesis of these B-cell lymphomas. In this context, ETS1 expression is more prominent in the ABC subtype while FLI1 dominates in GC DLBCL [55]. ETS1 interacts with and inhibits PRDM1 which plays an important role in plasma cell development [56]. Accordingly, downregulation of ETS1 and FLI1 is required for the final differentiation of this type of B-cells [57–59]. This relationship may be of relevance for HL because the tumor cells share some features with plasma cells [60,61]. Finally, the ETS factors ETS1, FLI1 and GAPBA interact with PAX5, demonstrating an additional regulatory mode of these factors to control B-cell differentiation processes [62]. Taken together, our data highlight that ETS factors are important players in B-cell differentiation and show that their deregulation contributes to the development of lymphoma. Understanding normal and abnormal regulatory networks controlled by ETS factors may serve to improve diagnostics and inform the design and evaluation of novel therapeutic approaches.

## Materials and methods

### Bioinformatic analyses

Expression data for normal hematopoietic cell types were obtained from Gene Ex-pression Omnibus (GEO, www.ncbi.nlm.nih.gov), using expression profiling dataset GSE56315 [63], and RNA-seq datasets GSE69239, GSE107011, GSE112591 and GSE90834 [64–67]. Gene expression profiling data from HL patients were examined using datasets GSE12453 and GSE39134 [68,69]. Applied cut offs were used as described in previous studies [5–8]. For screening of cell lines we exploited RNA-sequencing data from 100 leukemia/lymphoma cell lines (termed LL-100), available at ArrayExpress (www.ebi.ac.uk/arrayexpress) via E-MTAB-7721 [27]. LL-100 expression data were visualized using the online tool DSMZCellDive [70].

Gene set annotation analysis was performed using the online tool DAVID (provided by the Laboratory of Human Retrovirology and Immunoinformatics, www.david.abcc.ncifcrf.gov) [71]. For screening of TF binding sites we used the UCSC genome browser (www.genome.cse.ucsc.edu). ChIP-seq data were obtained from the ENCODE project and analyzed using the Integrative Genomics Viewer (provided by the Broad Institute, www.broadinstitute.org/data-software-and-tools). Expression profiling data from siRNA-treated SUP-HD1 cells were generated at the Genome Analytics Facility (Helmholtz Centre for Infection Research, Braunschweig, Germany) using HG U133 Plus 2.0 gene chips (Affymetrix) and are available at BioStudies (www.ebi.ac.uk/biostudies) via S-BSST1027. After RMA-background correction and quantile normalization of the spot intensities, profiling data were expressed as ratios of sample means and subsequently log2 transformed. Data processing was performed via R/Bioconductor using public limma and affy packages.

### Cell lines and treatments

Cell lines are held by the DSMZ (Braunschweig, Germany) and cultivated as de-scribed previously [72]. All cell lines had been authenticated and tested negative for mycoplasma infection. Modification of gene expression levels was performed using gene specific siRNA oligonucleotides with reference to AllStars negative Control siRNA (siCTR) obtained from Qiagen (Hilden, Germany). SiRNAs (80 pmol) were transfected into 1x10^6^ cells by electroporation using the EPI-2500 impulse generator (Fischer, Heidelberg, Germany) at 350 V for 10 ms. Electroporated cells were harvested after 20 h cultivation. Cell lines were treated with 20 ng/ml bone morphogenetic protein (BMP)2, BMP4 (R&D Systems, Wiesbaden, Germany), 10 μM dorsomorphin (Calbiochem, Darmstadt, Germany) dissolved in DMSO, and harvested after 20 h cultivation.

Proliferation and apoptosis were analyzed by live-cell-imaging using the IncuCyte S3 Live-Cell Analysis System and the associated software package Cell-by-Cell (Essen Bioscience, Essen, Germany). For detection of apoptotic cells, we used the IncuCyte Caspase-3/7 Green Apoptosis Assay diluted at 1:2,000 (Essen Bioscience). These experiments were performed twice with fourfold parallel tests. Statistical analyses were performed for the last time point of the experiments using Student´s T-Test. Standard deviations were indicated as error bars and calculated by the software.

### Polymerase chain-reaction (PCR) analyses

Total RNA was extracted from cultivated cell lines using TRIzol reagent (Invitrogen, Darmstadt, Germany). Primary human total RNA was purchased from Biochain/BioCat (Heidelberg, Germany). cDNA was synthesized using 5 μg RNA, random priming and Superscript II (Invitrogen). Real time quantitative (RQ)-PCR analysis was performed using the 7500 Real-time System and commercial buffer and primer sets (Applied Bio-systems/Life Technologies, Darmstadt, Germany). For normalization of expression levels, we quantified the transcripts of TATA box binding protein (TBP). Quantitative analyses were performed as biological replicates and measured in triplicate. Standard deviations are presented in the figures as error bars. Statistical significance was assessed by Student´s T-Test (two-tailed) and the calculated p-values indicated by asterisks (* p<0.05, ** p<0.01, *** p<0.001, n.s. not significant).

### Protein analysis

Western blots were generated by the semi-dry method. Protein lysates from cell lines were prepared using SIGMAFast protease inhibitor cocktail (Sigma, Taufkirchen, Germany). Proteins were transferred onto nitrocellulose membranes (Bio-Rad, Munich, Germany) and blocked with 5% dry milk powder dissolved in phosphate-buffered-saline buffer (PBS). The following antibodies were used: alpha-Tubulin (Sigma, #T6199), ETV3 (LSBioSciences, Eching, Germany, #LS-C342337), RIOK2 (MyBioSource, San Diego, CA, USA, #MBS9418079). For loading control, blots were reversibly stained with Poinceau (Sigma) and detection of alpha-Tubulin (TUBA) performed thereafter. Secondary antibodies were linked to peroxidase for detection by West-ern-Lightning-ECL (Perkin Elmer, Waltham, MA, USA). Documentation was performed using the digital system ChemoStar Imager (INTAS, Göttingen, Germany).

Immuno-cytology was performed as follows: cells were spun onto slides and sub-sequently air-dried and fixed with methanol/acetic acid for 90 s. Antibodies were diluted 1:20 in PBS containing 5% BSA and incubated for 30 min. Washing was performed 3 times with PBS. Preparations were incubated with secondary antibody (diluted 1:100) for 20 min. After final washing cells were mounted for nuclear in Vectashield (Vector Laboratories, Burlingame, CA), containing DAPI. Documentation of subcellular protein localization was performed by an Axion A1 microscope using the Axiocam 208 color and software ZEN 3.3 blue edition (Zeiss, Göttingen, Ger-many).

### Genomic profiling analysis

For genomic profiling genomic cell line DNA was prepared by the Qiagen Gentra Puregene Kit (Qiagen). Labelling, hybridization and scanning of Cytoscan HD arrays was performed by the Genome Analytics Facility located at the Helmholtz Centre for Infection Research, according to the manufacturer´s protocols (Affymetrix, High Wycombe, UK). Data were interpreted using the Chromosome Analysis Suite software version 3.1.0.15 (Affymetrix) and copy number alterations determined accordingly.

## Conclusions

We have described physiological expression patterns of 19 ETS genes in lympho-poiesis, which we termed the lymphoid ETS-code. Exploiting that code for evaluation of ETS genes in HL patients revealed 12 deregulated members in this B-cell malignancy. Detailed analyses of ETV3, ETS1 and FLI1 in HL cell lines discovered deregulatory mechanisms and target genes. Thus, our study should contribute to the understanding of normal and abnormal gene regulatory networks in developing blood and immune cells, findings which may assist diagnostic and therapeutic advances.

## Supporting information

S1 Fig ETS gene activities in early hematopoiesis and T-cell development using RNA-seq dataset GSE69239. (TIF)

S2 Fig ETS gene activities in B-cell development using gene expression profiling dataset GSE56315. (TIF)

S3 Fig ETS gene activities in ILCs using RNA-seq dataset GSE112591, and in ILCP using dataset GSE90834. (TIF)

S4 Fig ETS gene activities in diverse hematopoietic entities using RNA-seq dataset GSE107011. (TIF)

S5 Fig ETS gene activities in lymphoma patients and developing B-cells using gene expression profiling dataset GSE12453. Abbreviations: Hodgkin lymphoma (HL), T-cell rich B-cell lymphoma (TCRBL), follicular lymphoma (FL), Burkitt lymphoma (BL), diffuse large B-cell lymphoma (DLBCL), and normal B-cells: Naive B-cells (N), memory B-cells (M), germinal center B-cells (GC), plasma cells (P).
(TIF)

S6 Fig Selected ETS gene expression in hematopoietic cell lines using RNA-seq dataset LL-100 (E-MTAB-7721). (TIF)

S7 Fig Presentation of potential TF-binding sites at CD19 (above) and CELF2 (below) obtained from the UCSC genome browser. Binding sites for ETS factor ELK1 are indicated.
(TIF)

S8 Fig Results calculated by the DAVID online tool for the top-1000 differentially expressed genes in SUP-HD1 treated for siRNA-mediated knockdown of ETV3. Arrows indicate interesting functions, including differentiation and BMP-signalling.
(TIF)

S9 Fig Copy number analysis of selected HL cell lines for chromosome 21 (above) and 19 (below). The genes MIR155HG and JAK2 are indicated.
(TIF)

S1 Table Gene expression profiling analysis of SUP-HD1 treated for siRNA-mediated knockdown of ETV3. (XLS)

S1 File (PDF)

## References

[1] Rothenberg E.V. Transcriptional control of early T and B cell developmental choices. Annu. Rev. Immunol. 2014, 32, 283–321. doi: 10.1146/annurev-immunol-032712-100024 24471430 PMC3994230

[2] Kucinski I.;Wilson N.K.; Hannah R.; Kinston S.J.; Cauchy P.; Lenaerts A.; et al. Interactions between lineage-associated transcription factors govern haematopoietic progenitor states. EMBO J. 2020, 39, e104983. doi: 10.15252/embj.2020104983 33103827 PMC7737608

[3] Lambert SA, Jolma A, Campitelli LF, Das PK, Yin Y, Albu M, et al. The human transcription factors. Cell. 2018;175(2):598–599. doi: 10.1016/j.cell.2018.09.045 30290144

[4] Wilson NK, Foster SD, Wang X, Knezevic K, Schütte J, Kaimakis P, et al. Combinatorial transcriptional control in blood stem/progenitor cells: genome-wide analysis of ten major transcriptional regulators. Cell Stem Cell. 2010;7(4):532–544. doi: 10.1016/j.stem.2010.07.016 20887958

[5] Nagel S. NKL-Code in normal and aberrant hematopoiesis. Cancers 2021, 13, 1961. doi: 10.3390/cancers13081961 33921702 PMC8073162

[6] Nagel S.; Pommerenke C.; Meyer C.; MacLeod R.A.F.; Drexler H.G. Establishment of the TALE-code reveals aberrantly activated homeobox gene PBX1 in Hodgkin lymphoma. PLoS ONE 2021, 16, e0246603. doi: 10.1371/journal.pone.0246603 33539429 PMC7861379

[7] Nagel S, Pommerenke C, Meyer C, MacLeod RAF. The hematopoietic TALE-code shows normal activity of IRX1 in myeloid progenitors and reveals ectopic expression of IRX3 and IRX5 in acute myeloid leukemia. Int J Mol Sci. 2022;23(6):3192. doi: 10.3390/ijms23063192 35328612 PMC8952210

[8] Nagel S, Meyer C. Establishment of the TBX-code reveals aberrantly activated T-box gene TBX3 in Hodgkin lymphoma. PLoS One. 2021;16(11):e0259674. doi: 10.1371/journal.pone.0259674 34807923 PMC8608327

[9] Sharrocks AD. The ETS-domain transcription factor family. Nat Rev Mol Cell Biol. 2001;2(11):827–837. doi: 10.1038/35099076 11715049

[10] Hollenhorst PC, McIntosh LP, Graves BJ. Genomic and biochemical insights into the specificity of ETS transcription factors. Annu Rev Biochem. 2011;80:437–471. doi: 10.1146/annurev.biochem.79.081507.103945 21548782 PMC5568663

[11] Leprince D, Gegonne A, Coll J, de Taisne C, Schneeberger A, Lagrou C, et al. A putative second cell-derived oncogene of the avian leukaemia retrovirus E26. Nature. 1983;306(5941):395–397. doi: 10.1038/306395a0 6316156

[12] Donaldson LW, Petersen JM, Graves BJ, McIntosh LP. Solution structure of the ETS domain from murine Ets-1: a winged helix-turn-helix DNA binding motif. EMBO J. 1996;15(1):125–134. 8598195 PMC449924

[13] Aravind L, Anantharaman V, Balaji S, Babu MM, Iyer LM. The many faces of the helix-turn-helix domain: transcription regulation and beyond. FEMS Microbiol Rev. 2005;29(2):231–262. doi: 10.1016/j.femsre.2004.12.008 15808743

[14] Cooper CD, Newman JA, Gileadi O. Recent advances in the structural molecular biology of Ets transcription factors: interactions, interfaces and inhibition. Biochem Soc Trans. 2014;42(1):130–138. doi: 10.1042/BST20130227 24450640 PMC3901394

[15] Wei GH, Badis G, Berger MF, Kivioja T, Palin K, Enge M, et al. Genome-wide analysis of ETS-family DNA-binding in vitro and in vivo. EMBO J. 2010;29(13):2147–2160. doi: 10.1038/emboj.2010.106 20517297 PMC2905244

[16] Verger A, Duterque-Coquillaud M. When Ets transcription factors meet their partners. Bioessays. 2002;24(4):362–370. doi: 10.1002/bies.10068 11948622

[17] Maroulakou IG, Bowe DB. Expression and function of Ets transcription factors in mammalian development: a regulatory network. Oncogene. 2000;19(55):6432–6442. doi: 10.1038/sj.onc.1204039 11175359

[18] Wheat W, Fitzsimmons D, Lennox H, Krautkramer SR, Gentile LN, McIntosh LP, et al. The highly conserved beta-hairpin of the paired DNA-binding domain is required for assembly of Pax-Ets ternary complexes. Mol Cell Biol. 1999;19(3):2231–2241. doi: 10.1128/MCB.19.3.2231 10022910 PMC84016

[19] Garvie CW, Hagman J, Wolberger C. Structural studies of Ets-1/Pax5 complex formation on DNA. Mol Cell. 2001;8(6):1267–1276. doi: 10.1016/s1097-2765(01)00410-5 11779502

[20] Seth A, Watson DK. ETS transcription factors and their emerging roles in human cancer. Eur J Cancer. 2005 Nov;41(16):2462–2478. doi: 10.1016/j.ejca.2005.08.013 16213704

[21] Nagel S, Pommerenke C, Meyer C, MacLeod RAF. NKL homeobox genes NKX2-3 and NKX2-4 deregulate megakaryocytic-erythroid cell differentiation in AML. Int J Mol Sci. 2021;22(21):11434. 34768865 10.3390/ijms222111434 PMC8583893

[22] Alaggio R, Amador C, Anagnostopoulos I, Attygalle AD, Araujo IBO, Berti E, et al. The 5th edition of the World Health Organization classification of haematolymphoid tumours: lymphoid neoplasms. Leukemia. 2022;36(7):1720–1748. doi: 10.1038/s41375-022-01620-2 35732829 PMC9214472

[23] Weniger MA, Küppers R. Molecular biology of Hodgkin lymphoma. Leukemia. 2021;35(4):968–981. doi: 10.1038/s41375-021-01204-6 33686198 PMC8024192

[24] Krenacs L.; Himmelmann A.W.; Quintanilla-Martinez L.; Fest T.; Riva A.; Wellmann A.; et al. Transcription factor B-cell-specific activator protein (BSAP) is differentially expressed in B cells and in subsets of B-cell lymphomas. Blood 1998, 92, 1308–1316. 9694719

[25] Bohle V.; Döring C.; Hansmann M.L.; Küppers R. Role of early B-cell factor 1 (EBF1) in Hodgkin lymphoma. Leukemia 2013, 27, 671–679. doi: 10.1038/leu.2012.280 23174882

[26] MacLeod RAF, Nagel S, Scherr M, Schneider B, Dirks WG, Uphoff CC, et al. Human leukemia and lymphoma cell lines as models and resources. Curr Med Chem. 2008;15(4):339–359. doi: 10.2174/092986708783497319 18288989

[27] Quentmeier H, Pommerenke C, Dirks WG, Eberth S, Koeppel M, MacLeod RAF, et al. The LL-100 panel: 100 cell lines for blood cancer studies. Sci Rep. 2019;9(1):8218. doi: 10.1038/s41598-019-44491-x 31160637 PMC6547646

[28] Stein H, Foss HD, Dürkop H, Marafioti T, Delsol G, Pulford K, et al. CD30(+) anaplastic large cell lymphoma: a review of its histopathologic, genetic, and clinical features. Blood. 2000;96(12):3681–3695. 11090048

[29] Schlegelberger B, Weber-Matthiesen K, Himmler A, Bartels H, Sonnen R, Kuse R, et al. 1994. Cytogenetic findings and results of combined immunophenotyping and karyotyping in Hodgkin’s disease. Leukemia 8:72–80. 8289502

[30] MacLeod RA, Spitzer D, Bar-Am I, Sylvester JE, Kaufmann M, Wernich A, et al. 2000. Karyotypic dissection of Hodgkin’s disease cell lines reveals ectopic subtelomeres and ribosomal DNA at sites of multiple jumping translocations and genomic amplification. Leukemia 14:1803–1814. doi: 10.1038/sj.leu.2401894 11021756

[31] Nagel S, Meyer C, Quentmeier H, Kaufmann M, Drexler HG, MacLeod RA. Chromothripsis in Hodgkin lymphoma. Genes Chromosomes Cancer. 2013;52(8):741–747. doi: 10.1002/gcc.22069 23630094

[32] Mathas S, Hinz M, Anagnostopoulos I, Krappmann D, Lietz A, Jundt F, et al. Aberrantly expressed c-Jun and JunB are a hallmark of Hodgkin lymphoma cells, stimulate proliferation and synergize with NF-kappa B. EMBO J. 2002;21(15):4104–4113. doi: 10.1093/emboj/cdf389 12145210 PMC126136

[33] Küppers R, Klein U, Schwering I, Distler V, Bräuninger A, Cattoretti G, et al. Identification of Hodgkin and Reed-Sternberg cell-specific genes by gene expression profiling. J Clin Invest. 2003;111(4):529–537. doi: 10.1172/JCI16624 12588891 PMC151925

[34] Gerbe A, Alame M, Dereure O, Gonzalez S, Durand L, Tempier A, et al. Systemic, primary cutaneous, and breast implant-associated ALK-negative anaplastic large-cell lymphomas present similar biologic features despite distinct clinical behavior. Virchows Arch. 2019;475(2):163–174. doi: 10.1007/s00428-019-02570-4 30953147

[35] Watanabe M, Sasaki M, Itoh K, Higashihara M, Umezawa K, Kadin ME, et al. JunB induced by constitutive CD30-extracellular signal-regulated kinase 1/2 mitogen-activated protein kinase signaling activates the CD30 promoter in anaplastic large cell lymphoma and reed-sternberg cells of Hodgkin lymphoma. Cancer Res. 2005;65(17):7628–7634. doi: 10.1158/0008-5472.CAN-05-0925 16140928

[36] Kluiver J, Poppema S, de Jong D, Blokzijl T, Harms G, Jacobs S, et al. BIC and miR-155 are highly expressed in Hodgkin, primary mediastinal and diffuse large B cell lymphomas. J Pathol. 2005;207(2):243–249. doi: 10.1002/path.1825 16041695

[37] Ghosh S, Raundhal M, Myers SA, Carr SA, Chen X, Petsko GA, et al. Identification of RIOK2 as a master regulator of human blood cell development. Nat Immunol. 2022;23(1):109–121. doi: 10.1038/s41590-021-01079-w 34937919

[38] Ciau-Uitz A, Wang L, Patient R, Liu F. ETS transcription factors in hematopoietic stem cell development. Blood Cells Mol Dis. 2013;51(4):248–255. doi: 10.1016/j.bcmd.2013.07.010 23927967

[39] Mesquita B, Lopes P, Rodrigues A, Pereira D, Afonso M, Leal C, et al. Frequent copy number gains at 1q21 and 1q32 are associated with overexpression of the ETS transcription factors ETV3 and ELF3 in breast cancer irrespective of molecular subtypes. Breast Cancer Res Treat. 2013;138(1):37–45. doi: 10.1007/s10549-013-2408-2 23329352

[40] Brown RA, Kwong BY, McCalmont TH, Ragsdale B, Ma L, Cheung C, et al. ETV3-NCOA2 in in-determinate cell histiocytosis: clonal translocation supports sui generis. Blood. 2015;126(20):2344–2345. doi: 10.1182/blood-2015-07-655530 26438513

[41] Belina ME, Kwock JT, Al-Rohil R, Fresco A. An atypical myelomonocytic cell infiltrate: use of next-generation sequencing to diagnose indeterminate cell histiocytosis. Am J Dermatopathol. 2022;44(7):529–531. doi: 10.1097/DAD.0000000000002167 35234186

[42] Klappacher GW, Lunyak VV, Sykes DB, Sawka-Verhelle D, Sage J, Brard G, et al. An induced Ets repressor complex regulates growth arrest during terminal macro-phage differentiation. Cell. 2002;109(2):169–180. doi: 10.1016/s0092-8674(02)00714-6 12007404

[43] Villar J, Cros A, De Juan A, Alaoui L, Bonte PE, Lau CM, et al. ETV3 and ETV6 enable monocyte differentiation into dendritic cells by repressing macrophage fate commitment. Nat Immunol. 2023;24(1):84–95. doi: 10.1038/s41590-022-01374-0 36543959 PMC9810530

[44] Bier E, De Robertis EM. EMBRYO DEVELOPMENT. BMP gradients: A paradigm for morphogen-mediated developmental patterning. Science. 2015 Jun 26;348(6242):aaa5838. doi: 10.1126/science.aaa5838 26113727

[45] Blank U, Karlsson S. The role of Smad signaling in hematopoiesis and translational hematology. Leukemia. 2011;25(9):1379–1388. doi: 10.1038/leu.2011.95 21566654

[46] Passa O, Tsalavos S, Belyaev NN, Petryk A, Potocnik AJ, Graf D. Compartmentalization of bone morphogenetic proteins and their antagonists in lymphoid progenitors and supporting microenvironments and functional implications. Immu-nology. 2011;134(3):349–359. doi: 10.1111/j.1365-2567.2011.03495.x 21978004 PMC3209574

[47] Huse K, Bakkebø M, Wälchli S, Oksvold MP, Hilden VI, Forfang L, et al. Role of Smad proteins in resistance to BMP-induced growth inhibition in B-cell lymphoma. PLoS One. 2012;7(10):e46117. doi: 10.1371/journal.pone.0046117 23049692 PMC3462182

[48] Nagel S, Ehrentraut S, Meyer C, Kaufmann M, Drexler HG, MacLeod RA. Repressed BMP signaling reactivates NKL homeobox gene MSX1 in a T-ALL subset. Leuk Lymphoma. 2015;56(2):480–491. doi: 10.3109/10428194.2014.924119 24844359

[49] Flavell JR, Baumforth KR, Wood VH, Davies GL, Wei W, Reynolds GM, et al. Down-regulation of the TGF-beta target gene, PTPRK, by the Epstein-Barr virus encoded EBNA1 contributes to the growth and survival of Hodgkin lymphoma cells. Blood. 2008;111(1):292–301. doi: 10.1182/blood-2006-11-059881 17720884

[50] Louafi F, Martinez-Nunez RT, Sanchez-Elsner T. MicroRNA-155 targets SMAD2 and modulates the response of macro-phages to transforming growth factor-{beta}. J Biol Chem. 2010;285(53):41328–41336. doi: 10.1074/jbc.M110.146852 21036908 PMC3009858

[51] Yoon J.S.J.; Wu M.K.; Zhu T.H.; Zhao H.; Cheung S.T.; Chamberlain T.C.; et al. Interleukin-10 control of pre-miR155 maturation involves CELF2. PLoS ONE 2020, 15, e0231639. doi: 10.1371/journal.pone.0231639 32324763 PMC7179890

[52] Overbeck BM, Martin-Subero JI, Ammerpohl O, Klapper W, Siebert R, Giefing M. ETS1 encoding a transcription factor involved in B-cell differentiation is recurrently deleted and down-regulated in classical Hodgkin’s lymphoma. Haematologica. 2012;97(10):1612–1614. doi: 10.3324/haematol.2012.061770 22581005 PMC3487565

[53] He K, Chen Z, Zhao J, He Y, Deng R, Fan X, et al. The role of microRNA-155 in glomerular endothelial cell injury induced by high glucose. Mol Biol Rep. 2022;49(4):2915–2924. doi: 10.1007/s11033-021-07106-1 35064409 PMC8924107

[54] Bonetti P, Testoni M, Scandurra M, Ponzoni M, Piva R, Mensah AA, et al. Deregulation of ETS1 and FLI1 contributes to the pathogenesis of diffuse large B-cell lymphoma. Blood. 2013;122(13):2233–2241. doi: 10.1182/blood-2013-01-475772 23926301

[55] Sartori G, Napoli S, Cascione L, Chung EYL, Priebe V, Arribas AJ, et al. ASB2 is a direct target of FLI1 that sustains NF-κB pathway activation in germinal center-derived diffuse large B-cell lymphoma. J Exp Clin Cancer Res. 2021;40(1):357. 34763718 10.1186/s13046-021-02159-3 PMC8582153

[56] John SA, Clements JL, Russell LM, Garrett-Sinha LA. Ets-1 regulates plasma cell differentiation by interfering with the activity of the transcription factor Blimp-1. J Biol Chem. 2008;283(2):951–962. doi: 10.1074/jbc.M705262200 17977828

[57] Bories JC, Willerford DM, Grévin D, Davidson L, Camus A, Martin P, et al. Increased T-cell apoptosis and terminal B-cell differentiation induced by inactivation of the Ets-1 proto-oncogene. Nature. 1995;377(6550):635–638. doi: 10.1038/377635a0 7566176

[58] Hauser J, Verma-Gaur J, Wallenius A, Grundström T. Initiation of antigen receptor-dependent differentiation into plasma cells by calmodulin inhibition of E2A. J Immunol. 2009;183(2):1179–1187. doi: 10.4049/jimmunol.0900455 19553523

[59] Zhang XK, Moussa O, LaRue A, Bradshaw S, Molano I, Spyropoulos DD, et al. The transcription factor Fli-1 modulates marginal zone and follicular B cell development in mice. J Immunol. 2008;181(3):1644–1654. doi: 10.4049/jimmunol.181.3.1644 18641300 PMC2504761

[60] Buettner M, Greiner A, Avramidou A, Jäck HM, Niedobitek G. Evidence of abortive plasma cell differentiation in Hodgkin and Reed-Sternberg cells of classical Hodgkin lymphoma. Hematol Oncol. 2005;23(3–4):127–132. doi: 10.1002/hon.764 16342298

[61] Seitz V, Thomas PE, Zimmermann K, Paul U, Ehlers A, Joosten M, et al. Classical Hodgkin’s lymphoma shows epigenetic features of abortive plasma cell differentiation. Haematologica. 2011;96(6):863–870. doi: 10.3324/haematol.2010.031138 21393330 PMC3105648

[62] Maier H, Ostraat R, Parenti S, Fitzsimmons D, Abraham LJ, Garvie CW, et al. Requirements for selective recruitment of Ets proteins and activation of mb-1/Ig-alpha gene transcription by Pax-5 (BSAP). Nucleic Acids Res. 2003;31(19):5483–5489. doi: 10.1093/nar/gkg785 14500810 PMC206479

[63] Dybkær K, Bøgsted M, Falgreen S, Bødker JS, Kjeldsen MK, Schmitz A, et al. Diffuse large B-cell lymphoma classification system that associates normal B-cell subset phenotypes with prognosis. J Clin Oncol. 2015;33(12):1379–1388. doi: 10.1200/JCO.2014.57.7080 25800755 PMC4397280

[64] Casero D, Sandoval S, Seet CS, Scholes J, Zhu Y, Ha VL, et al. Long non-coding RNA profiling of human lymphoid progenitor cells reveals transcriptional divergence of B cell and T cell lineages. Nat Immunol. 2015;16(12):1282–1291. doi: 10.1038/ni.3299 26502406 PMC4653072

[65] Monaco G, Lee B, Xu W, Mustafah S, Hwang YY, Carré C, et al. RNA-seq signatures normalized by mRNA abundance allow absolute deconvolution of human immune cell types. Cell Rep. 2019;26(6):1627–1640. doi: 10.1016/j.celrep.2019.01.041 30726743 PMC6367568

[66] Li S, Morita H, Sokolowska M, Tan G, Boonpiyathad T, Opitz L, et al. Gene expression signatures of circulating human type 1, 2, and 3 innate lymphoid cells. J Allergy Clin Immunol. 2019;143(6):2321–2325. doi: 10.1016/j.jaci.2019.01.047 30825467

[67] Lim AI, Li Y, Lopez-Lastra S, Stadhouders R, Paul F, Casrouge A, et al. Systemic human ILC precursors provide a substrate for tissue ILC differentiation. Cell. 2017;168(6):1086–1100. doi: 10.1016/j.cell.2017.02.021 28283063

[68] Brune V, Tiacci E, Pfeil I, Döring C, Eckerle S, van Noesel CJ, et al. Origin and pathogenesis of nodular lymphocyte-predominant Hodgkin lymphoma as revealed by global gene expression analysis. J Exp Med. 2008;205(10):2251–2268. doi: 10.1084/jem.20080809 18794340 PMC2556780

[69] Steidl C, Diepstra A, Lee T, Chan FC, Farinha P, Tan K, et al. Gene expression profiling of microdissected Hodgkin Reed-Sternberg cells correlates with treatment outcome in classical Hodgkin lymphoma. Blood. 2012;120(17):3530–3540. doi: 10.1182/blood-2012-06-439570 22955918

[70] Koblitz J, Dirks WG, Eberth S, Nagel S, Steenpass L, Pommerenke C. DSMZCellDive: Diving into high-throughput cell line data. F1000Res. 2022;11:420. doi: 10.12688/f1000research.111175.2 35949917 PMC9334839

[71] Huang D.W.; Sherman B.T.; Tan Q.; Collins J.R.; Alvord W.G.; Roayaei J.; et al. DAVID Gene functional classification tool: A novel biological module-centric algorithm to functionally analyze large gene list. Genome Biol. 2007, 8, R183. 17784955 10.1186/gb-2007-8-9-r183 PMC2375021

[72] Drexler H.G. Guide to leukemia-lymphoma cell lines, 2nd ed.; DSMZ: Braunschweig, Germany, 2010.

