# Supplementary figures and images for: Establishment of the lymphoid ETS-code reveals deregulated ETS genes in Hodgkin lymphoma
Source: PLoS One. 2023 Jul 10;18(7):e0288031. doi: 10.1371/journal.pone.0288031 (PMC10332584; doi:10.1371/journal.pone.0288031)

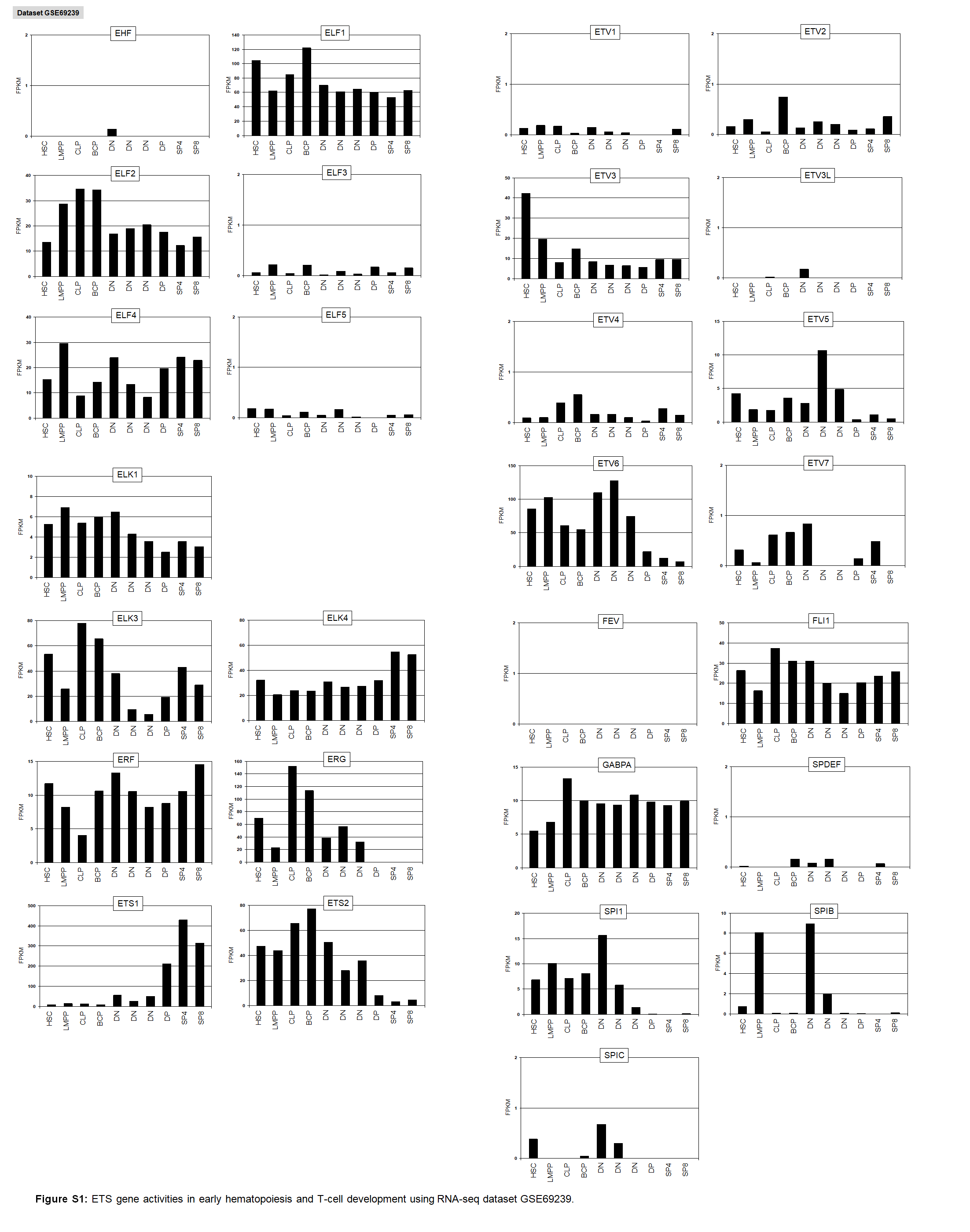

Supplement: S1 Fig — (TIF) [file pone.0288031.s001.tif]

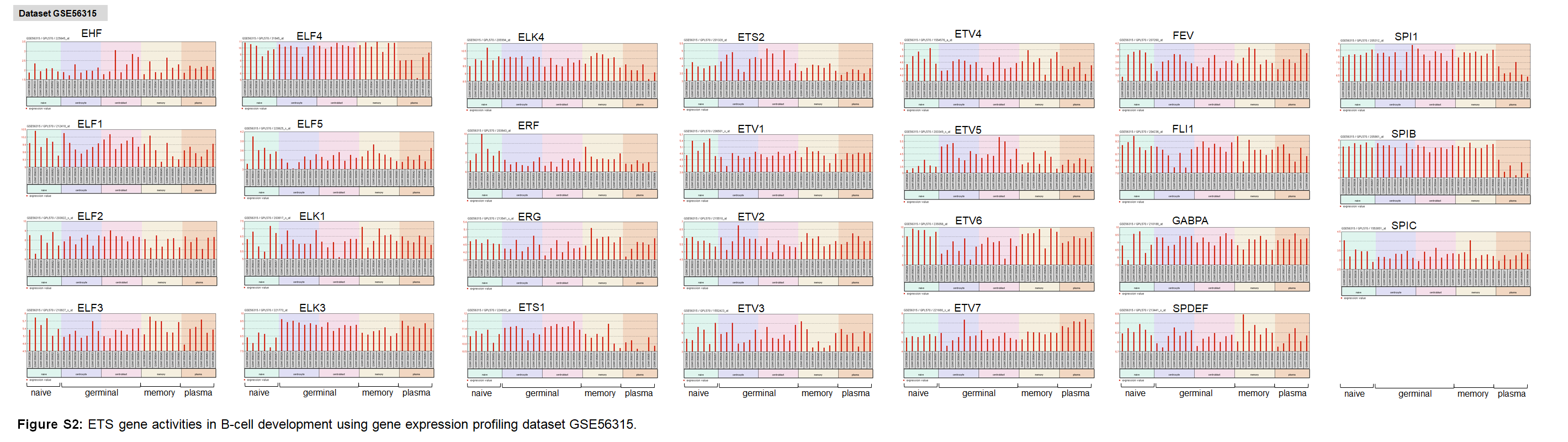

Supplement: S2 Fig — (TIF) [file pone.0288031.s002.tif]

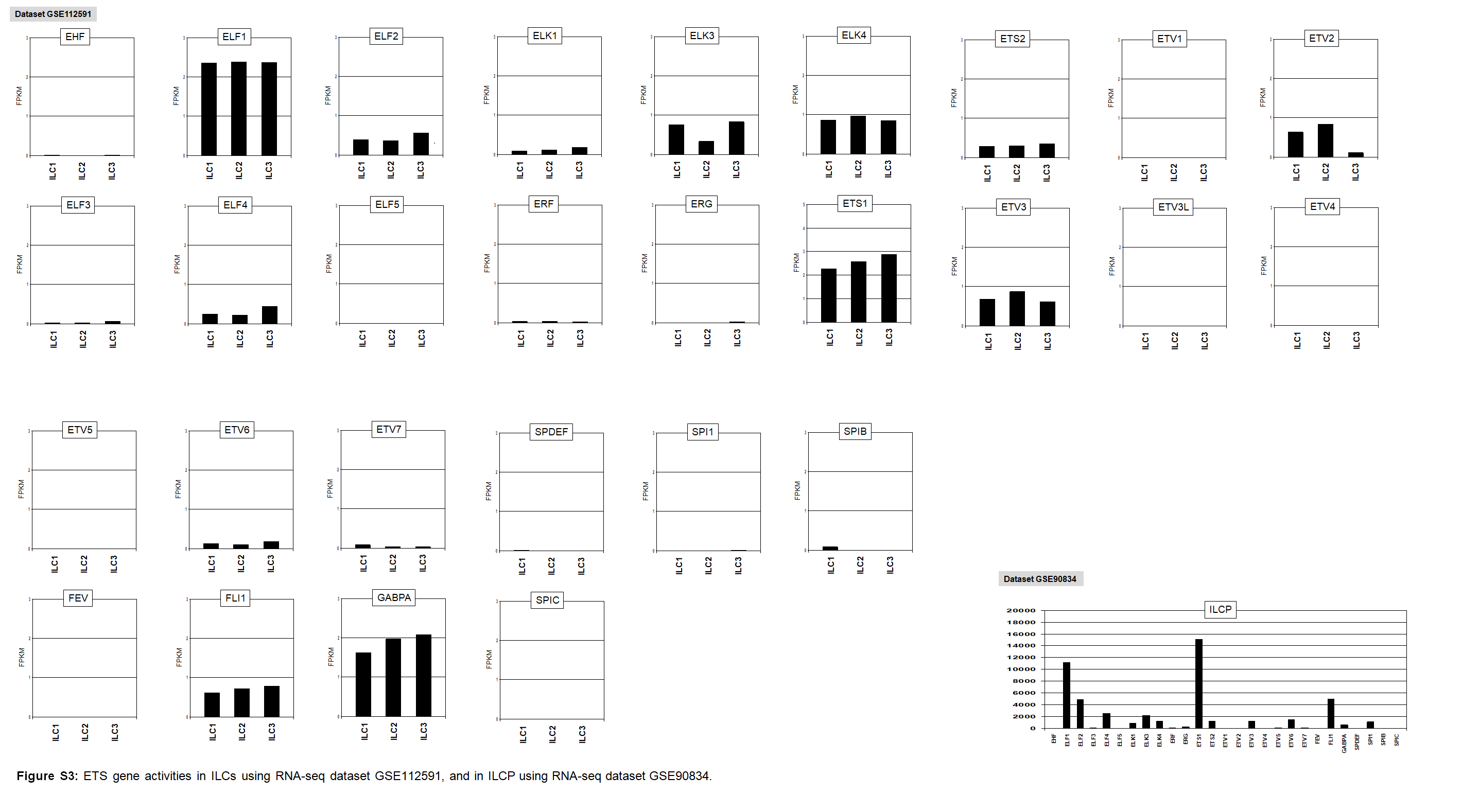

Supplement: S3 Fig — (TIF) [file pone.0288031.s003.tif]

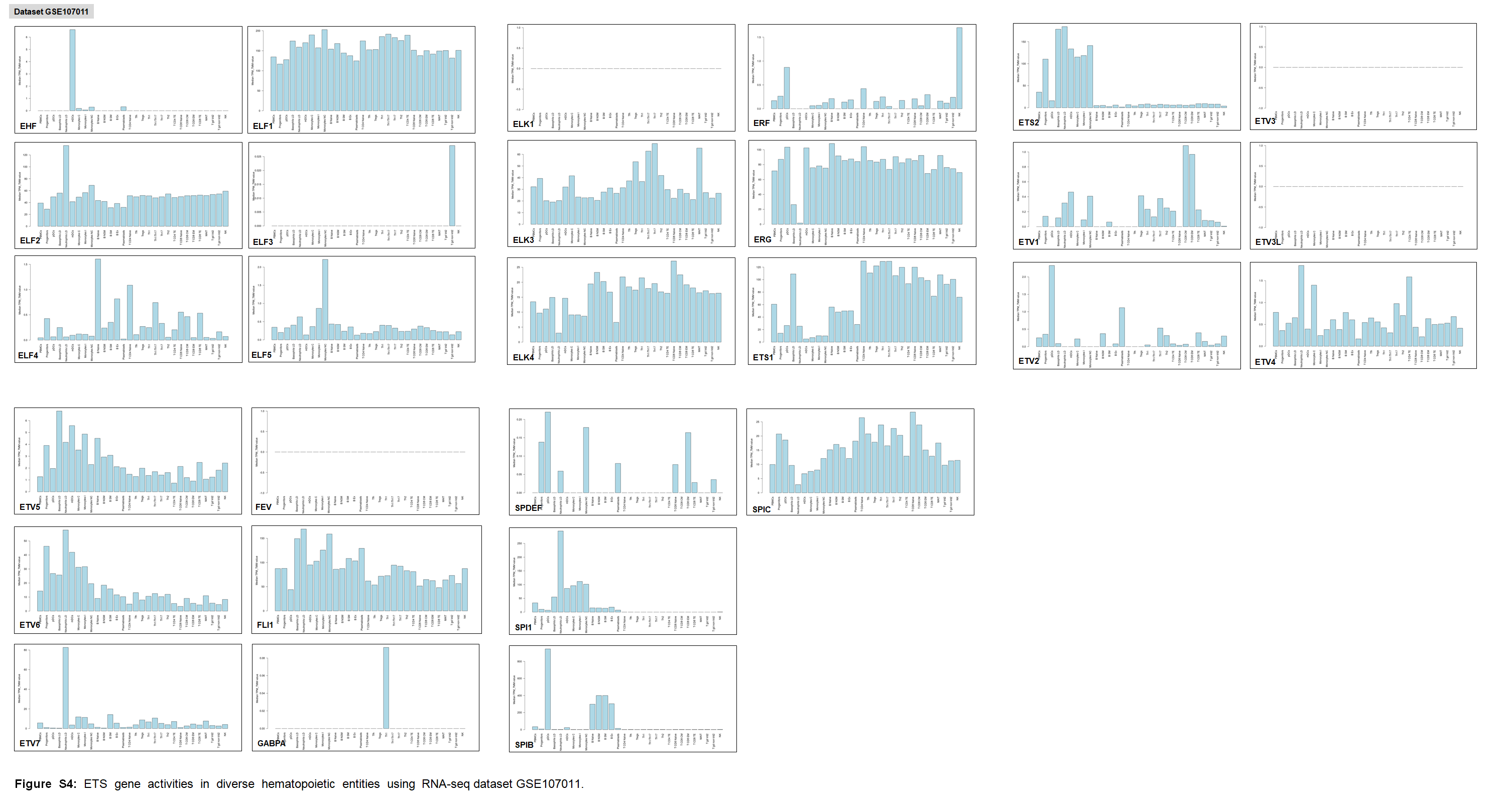

Supplement: S4 Fig — (TIF) [file pone.0288031.s004.tif]

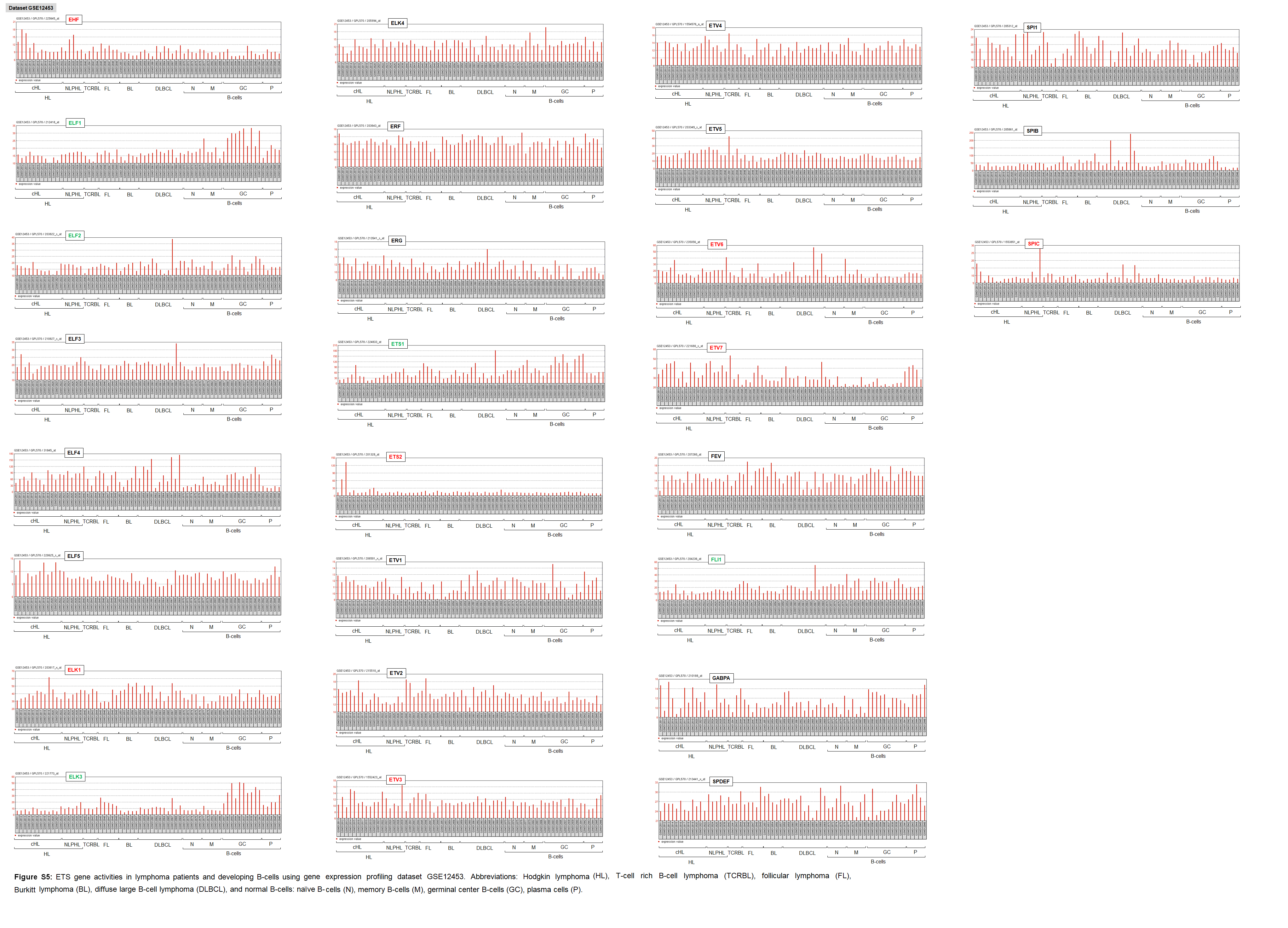

Supplement: S5 Fig — Abbreviations: Hodgkin lymphoma (HL), T-cell rich B-cell lymphoma (TCRBL), follicular lymphoma (FL), Burkitt lymphoma (BL), diffuse large B-cell lymphoma (DLBCL), and normal B-cells: Naive B-cells (N), memory B-cells (M), germinal center B-cells (GC), plasma cells (P). (TIF) [file pone.0288031.s005.tif]

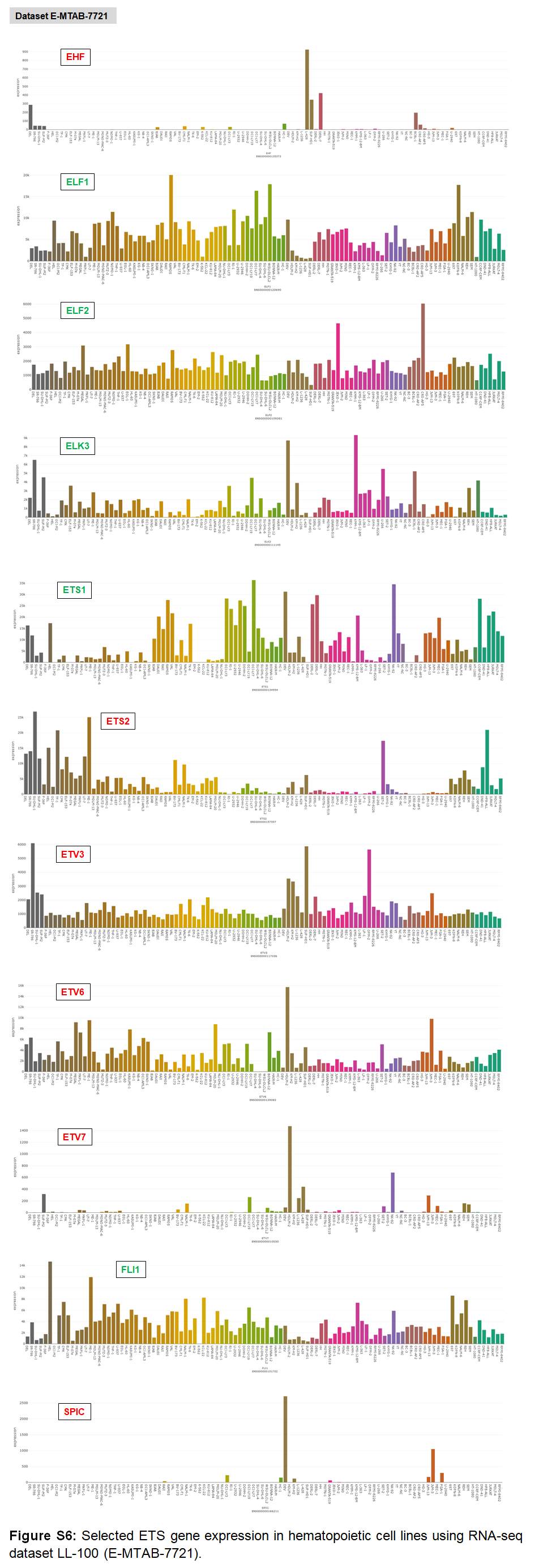

Supplement: S6 Fig — (TIF) [file pone.0288031.s006.tif]

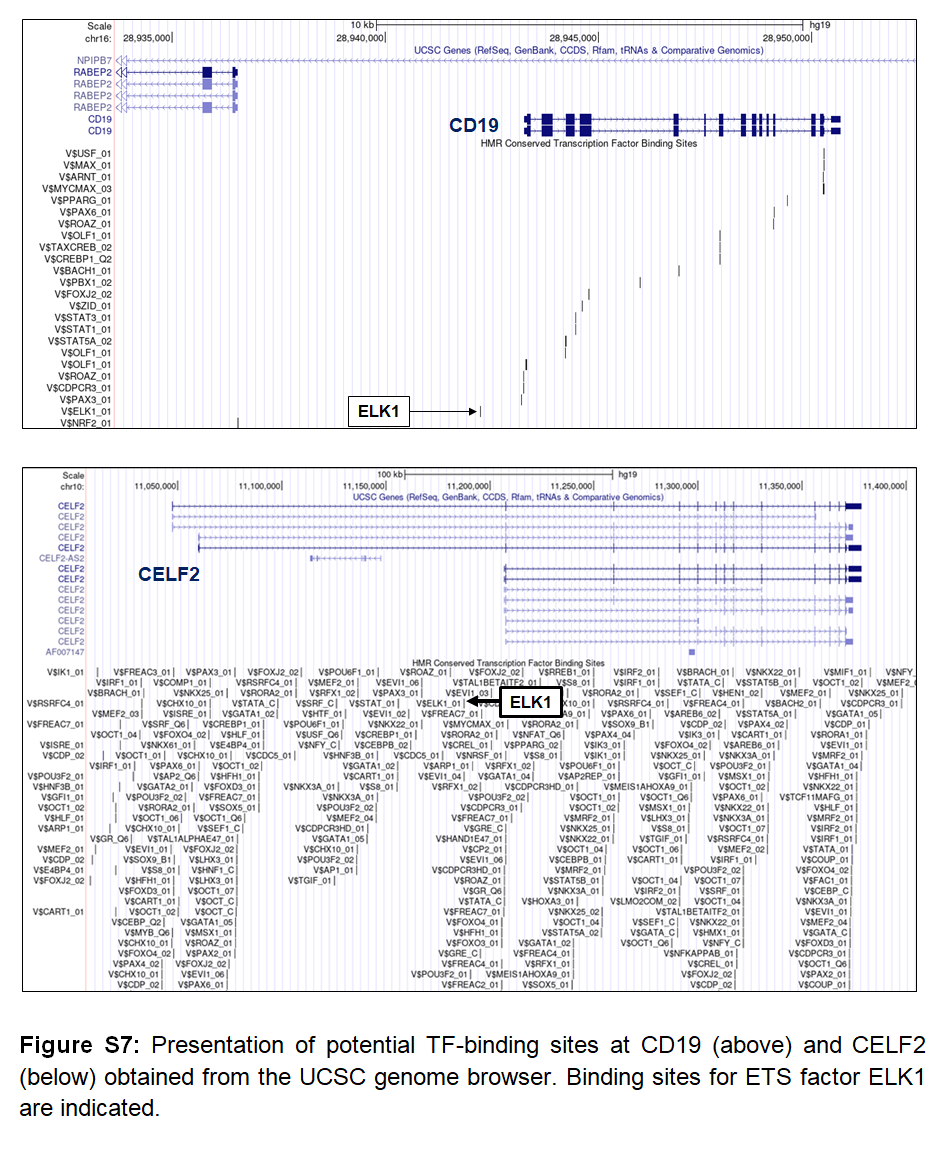

Supplement: S7 Fig — Binding sites for ETS factor ELK1 are indicated. (TIF) [file pone.0288031.s007.tif]

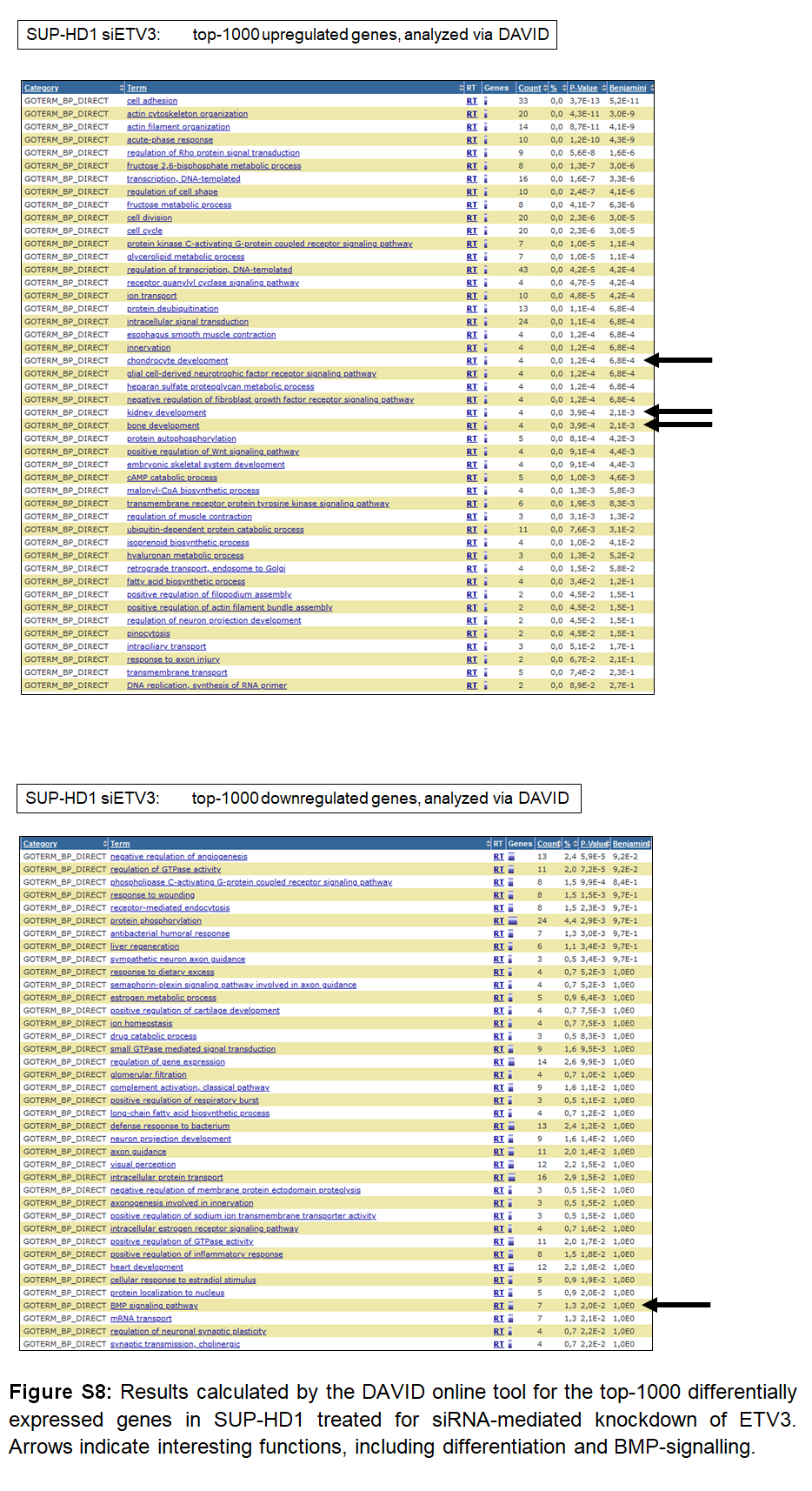

Supplement: S8 Fig — Arrows indicate interesting functions, including differentiation and BMP-signalling. (TIF) [file pone.0288031.s008.tif]

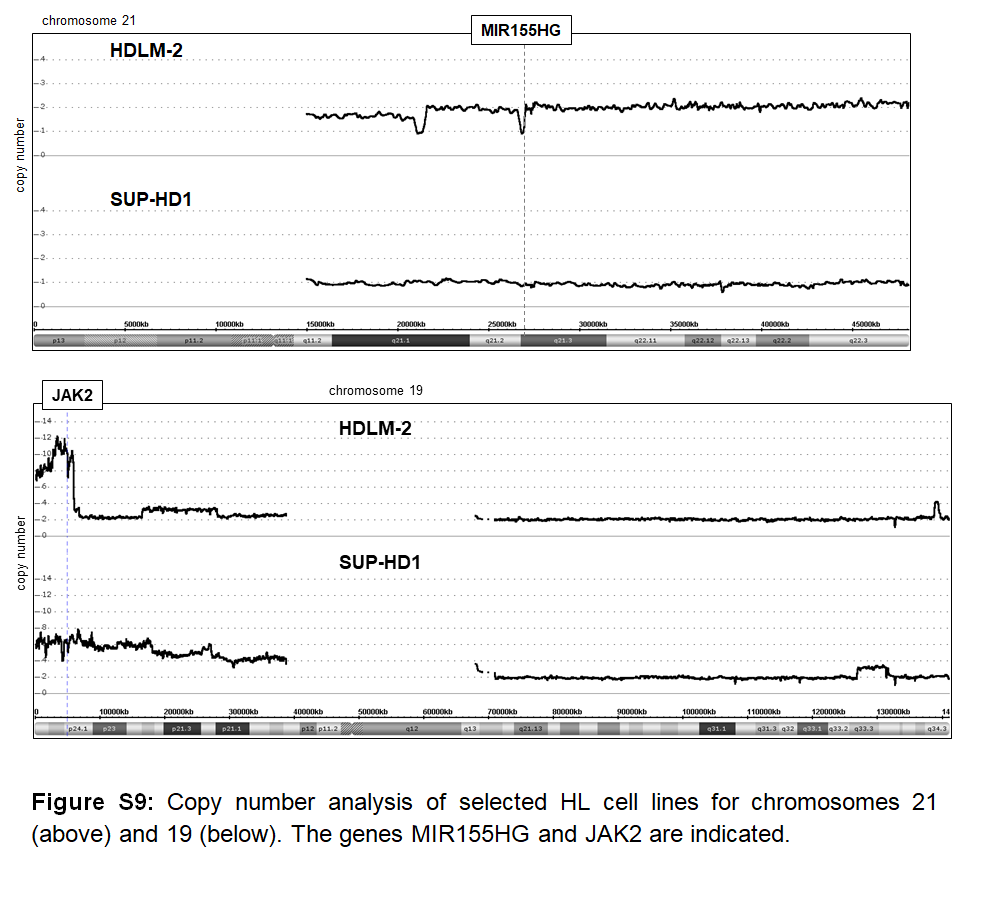

Supplement: S9 Fig — The genes MIR155HG and JAK2 are indicated. (TIF) [file pone.0288031.s009.tif]

Figure 2D

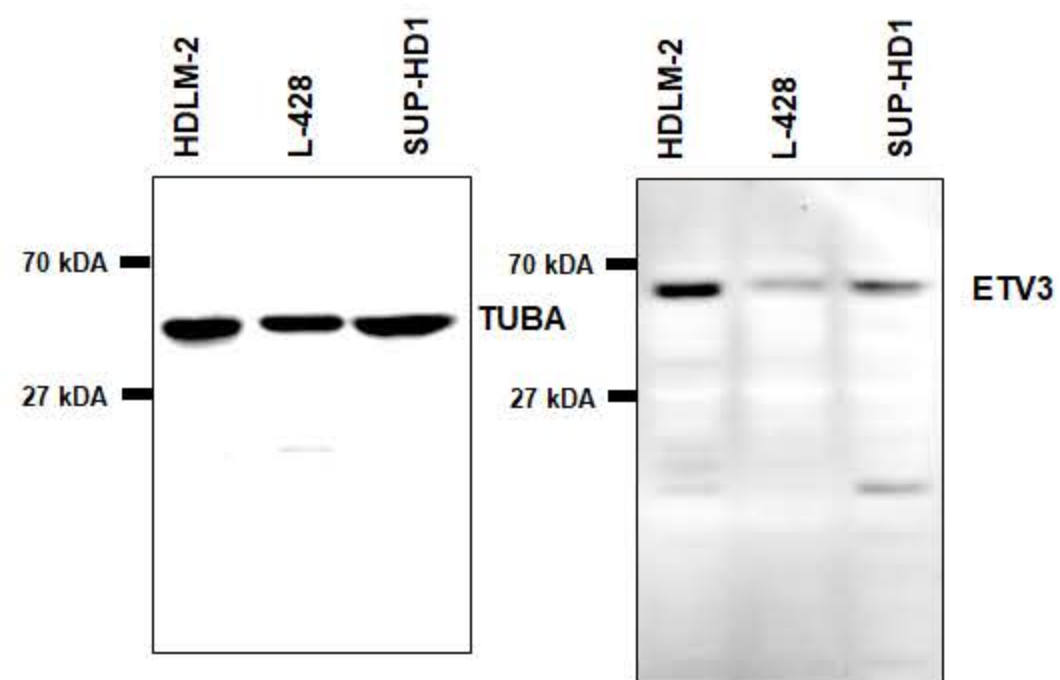

Figure 3E

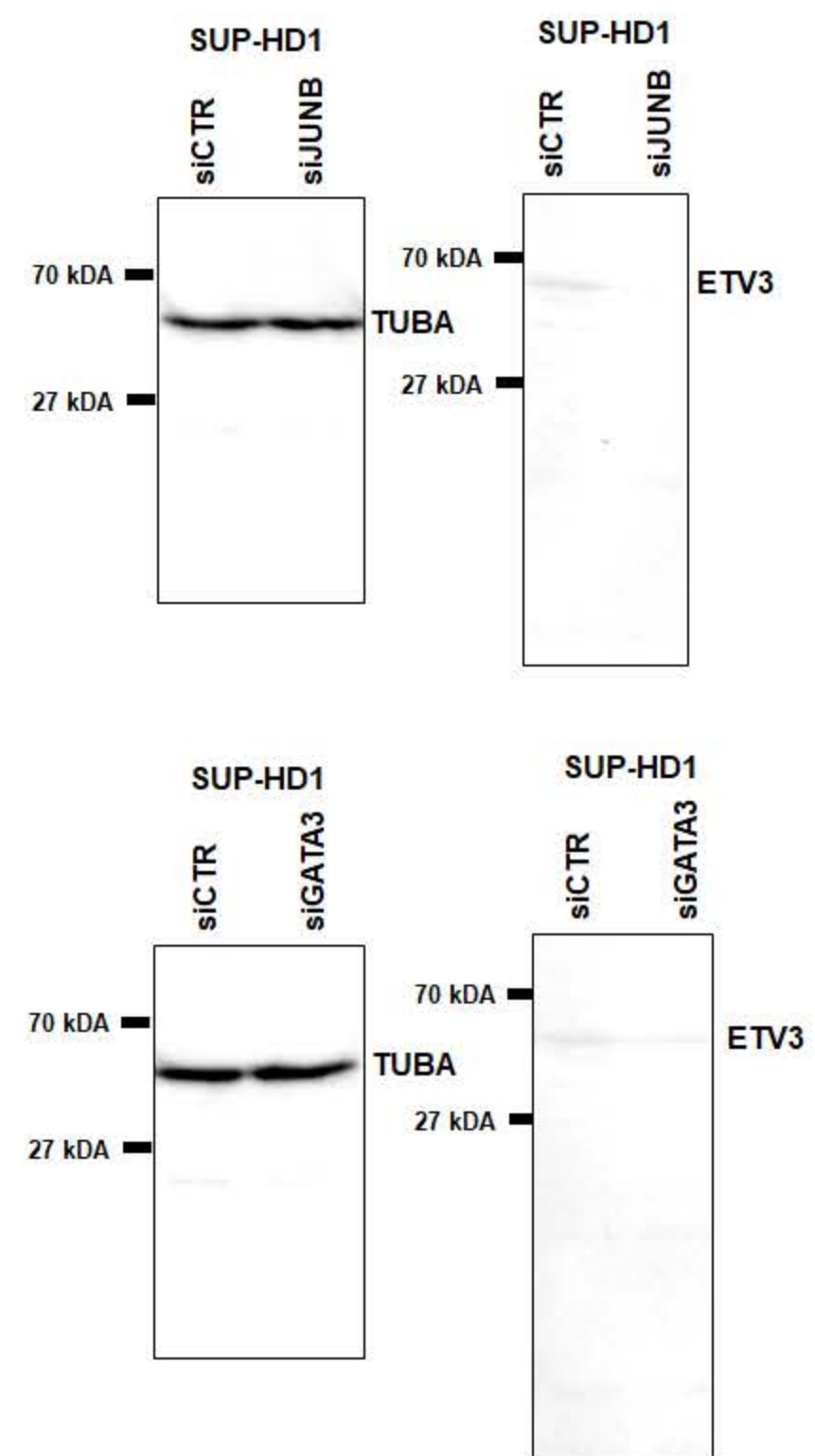

Figure 4A

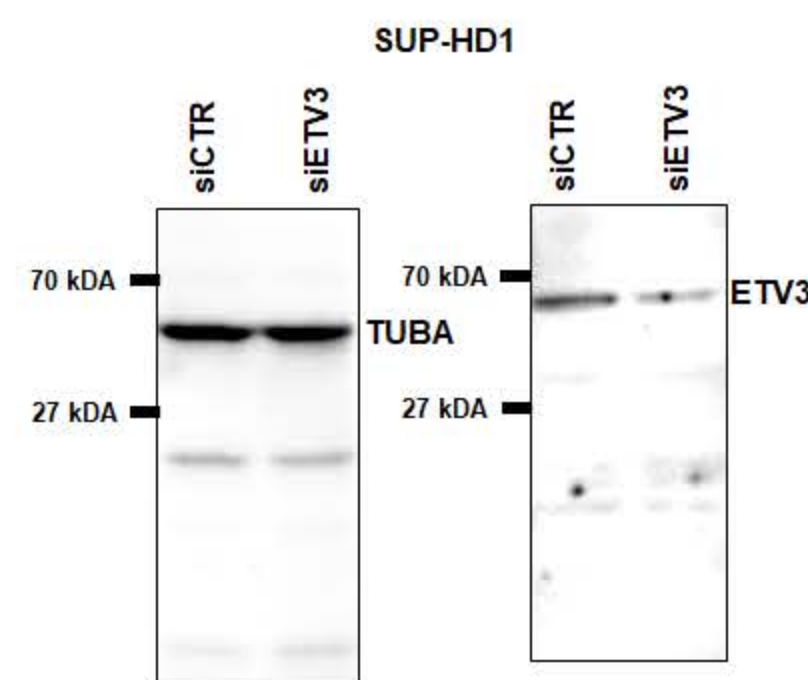

Figure 7B

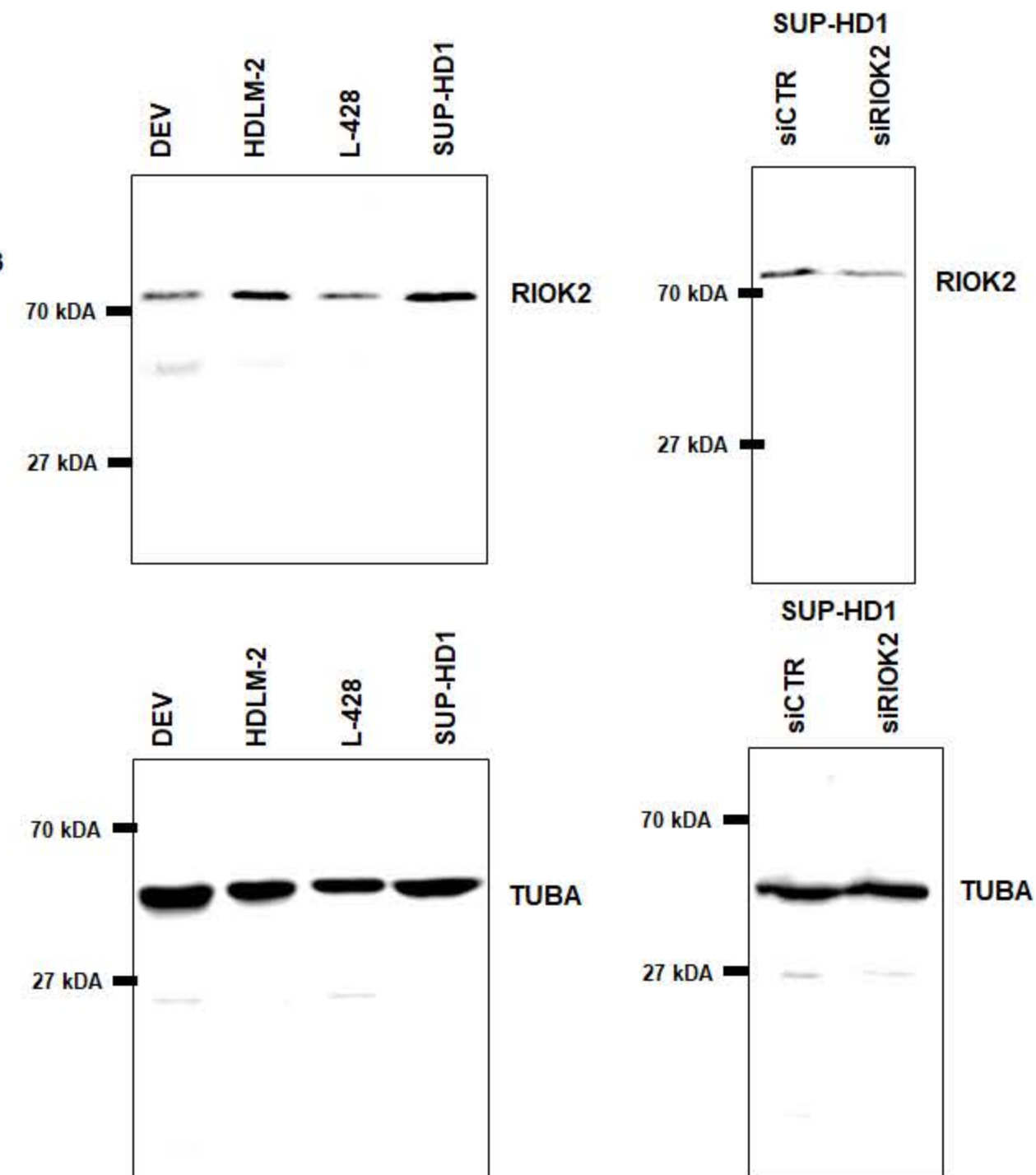

Figure 7G

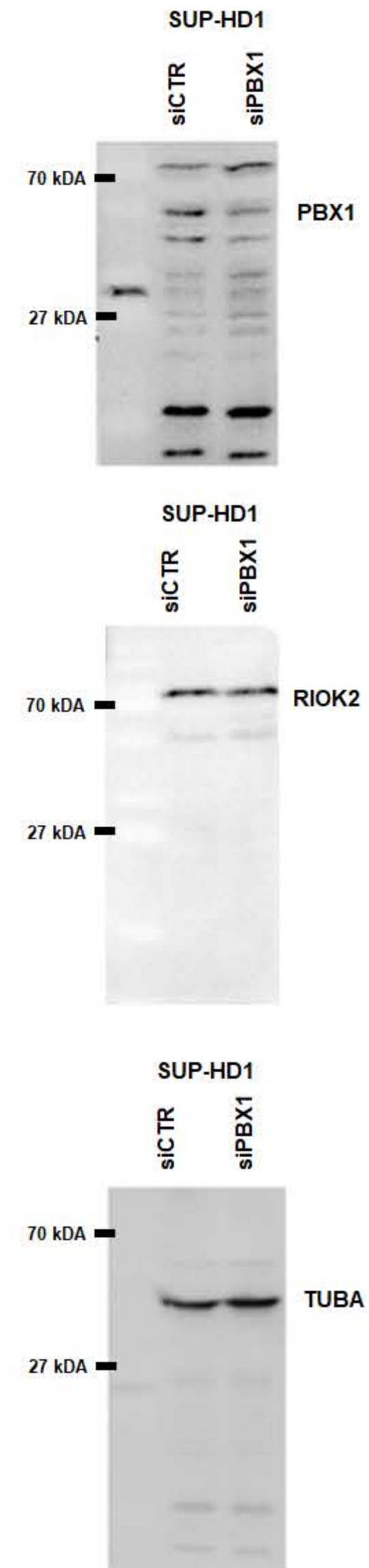

Supplement: S1 File — (PDF) [file pone.0288031.s011.pdf]
